# An Efficient Method for the Preparation of Sulfonamides from Sodium Sulfinates and Amines

**DOI:** 10.1002/open.202200097

**Published:** 2022-08-25

**Authors:** Haiying Tian, Ruiyan Li, Fang Guo, Xiuling Chen

**Affiliations:** ^1^ Department of Pharmacy Changzhi Medical College 046000 Changzhi P. R. China; ^2^ Department of Materials Science and Engineering Jinzhong University 030619 Jinzhong P. R. China

**Keywords:** sulfonamides, sodium sulfinates, NH_4_I, green protocols, metal-free

## Abstract

Sulfonamides have a special role on medicine due to their broad biological activities, as bacterial infections, diabetes mellitus, oedema, hypertension prevention and treatment. In addition, sulfonamides are also useful in herbicides and pesticides. Herein, we communicate an efficient strategy for the preparation of sulfonamides via NH_4_I‐mediated amination of sodium sulfinates. This new method provides a general and environmentally friendly access to sulfonamide compounds and tolerates a wide range of functional groups.

## Graphical abstract

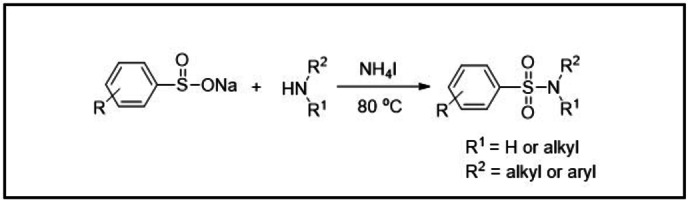

## Introduction

Containing S−N bonds compounds are a class of important structural motifs, which are of crucial relevance for biological activities, pesticide activities and material properties.[^1^, ^2^] Among all S−N compounds, sulfonamides are an important functional group in pharmaceutical interesting molecules and biologically active compounds.[^3^, ^4^] Some have proved to be useful as antibacterial, anticancer, antitumor, anti‐inflammatory and HIV protease inhibitory activities.[^5^, ^6^, ^7^, ^8^] In additional, sulfonamides are served as amine protecting group due to their easy deprotection under mild conditions.[^9^, ^10^] So, it is important to develop efficient method for the synthesis of sulfonamides. Conventional procedures for preparation of sulfonamides depend on the corrosive sulfonyl chlorides with amines in the presence of strong base or add acylating catalyst (i. e., DMAP) to the system.[^11^, ^12^] Recently, many novel routes for the synthesis of sulfonamides are developed.[^13^, ^14^, ^15^, ^16^, ^17^, ^18^, ^19^, ^20^, ^21^, ^22^, ^23^, ^24^, ^25^, ^26^, ^27^, ^28^, ^29^, ^30^, ^31^, ^32^, ^33^, ^34^, ^35^, ^36^, ^37^, ^38^] Metal catalyzed coupling reactions of sulfonamides and sodium sulfinates with organohalides or boronic acids provide an efficient system for the construction of sulfonamides.[^13^, ^14^, ^15^, ^16^, ^17^, ^18^, ^19^, ^20^] An attractive protocol for the synthesis of sulfonamides from oxidative coupling thiols or sulfinate salts with amines has also been reported.^[13]^ Currently, many improvements are focused on iodine‐mediated or iodine‐catalyzed reactions and transformation due to iodine is cheap, readily available and eco‐friendly.[^39^, ^40^, ^41^] I_2_‐mediated sodium sulfinates with amines reactions provide an efficient method to build up sulfonamides.[^25^, ^26^] Incorporation of sulfur dioxide unit into molecules reaction has emerged as an effective tool for the formation of sulfonamide. Using this strategy, *N*‐aminosulfonamides are synthesized via metal‐catalyzed coupling aryl iodides, boronic acids, with sulfur dioxide, or DABCO.[^28^, ^29^, ^30^, ^31^, ^32^, ^33^, ^34^, ^35^, ^36^, ^37^, ^38^] Although great progress has been achieved on the synthesis of sulfonamides,[^42^, ^43^, ^44^, ^45^, ^46^, ^47^, ^48^, ^49^, ^50^] some drawbacks still exist in the current methods, such as toxic metal catalysts, harsh reaction conditions or additional additives are contained in the reaction system and limit its synthetic utility.

**Scheme 1:**
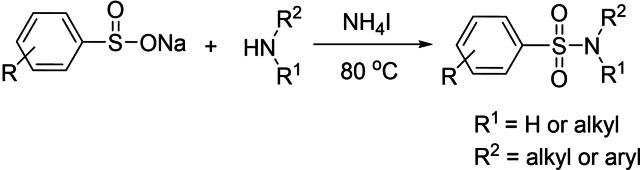
The route to the synthesis of sulfonamides.

Herein we report an efficient NH_4_I‐mediated reaction of sodium sulfinates and amines for preparation of sulfonamides (Scheme 1). This method shows good substrate scope (primary amines, secondary amines and imidazole, pyrazole, benzimidazole, indazole) and provides a variety of sulfonamide products in reasonable to excellent yields. As an alternative method to produce sulfonamides, the protocol has great applicability.

## Results and Discussion

Initially, the substrates of sodium *p*‐toluenesulfinate (**1 a**) and *n*‐propylamine (**2 a**) were carried out in the presence of NH_4_I for optimization of the reaction conditions and the results are compiled in Table 1. The sulfonamide **3 a** was obtained in 20 % yield in ethyl acetate (Table 1, entry 1). Encouraged by the result, various solvents (Table 1, entries 2–11) were screened and CH_3_CN was as a viable alternative to other solvents, the sulfonamide **3 a** was afforded in 85 % yield (Table 1, entry 11). The reaction was sensitive to temperature, for example only trace **3 a** was observed when the reaction was performed at 25 °C (Table 1, entry 12). Screening a range of additives NH_4_Cl, PhI(OAc)_2_, NaI, KI and I_2_, the results revealed that other reagents were ineffective for this transformation (Table 1, entries 13–17), the reaction was not proceeded in the absence of NH_4_I (Table 1, entry 18). Thus, the optimized conditions for this amination sodium sulfinates for the preparation of sulfonamides were summarized as follows: **1 a** (0.20 mmol), **2 a** (0.30 mmol), NH_4_I (0.2 mmol) in CH_3_CN (2 mL) at 80 °C for 12 h.

**Table 1:** Optimization of the reaction conditions.^[a]^

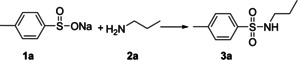			
Entry	Solvent	Additive	Yield of **3 a** ^[b]^
1	Ethyl acetate	NH_4_I	20
2	C_2_H_5_OH	NH_4_I	trace
3	THF	NH_4_I	25
4	1,4‐dioxane	NH_4_I	trace
5	DMF	NH_4_I	trace
6	DCE	NH_4_I	45
7	CH_2_Cl_2_	NH_4_I	20
8	Toluene	NH_4_I	55
9	CCl_4_	NH_4_I	45
10	*n*‐hexane	NH_4_I	40
11	CH_3_CN	NH_4_I	85
12^[c]^	CH_3_CN	NH_4_I	trace
13	CH_3_CN	NH_4_Cl	trace
14	CH_3_CN	PhI(OAc)_2_	40
15	CH_3_CN	NaI	56
16	CH_3_CN	KI	62
17	CH_3_CN	I_2_	55
18	CH_3_CN	–	trace

[a] Reaction conditions: **1 a** (0.2 mmol), **2 a** (0.3 mmol), additive (0.2 mmol), solvent (2 mL), 80 °C, under air in Schlenk tube for 12 h. [b] GC yield. [c] 25 °C.

The substrate scope of the present amination sodium sulfinates for the preparation of sulfonamides was further investigated and the results are summarized in Table 2. As shown, sodium *p*‐toluenesulfinate **1 a** reacted readily with both primary amines and secondary amines, furnishing the corresponding sulfonamides from moderate to excellent yields. For primary amines, *n*‐propylamine (**2 a**), *n*‐butylamine (**2 b**), *iso*‐butylamine (**2 c**), *iso*‐propylamine (**2 d**) reacted readily with sodium *p*‐toluenesulfinate to give the corresponding sulfonamides **3 a**–**3 d** in moderate yields (Table 2, entries 1–4). While, *tert*‐butylamine (**2 e**) was less efficiently and the yield of **3 e** was decreased to 45 % (Table 2, entry 5). It was worth mentioning that benzylamine **2 f** could be effective to afford the corresponding sulfonamide in 75 % yield (Table 2, entry 6). Secondary animes and cyclic amines such as diethylamine **2 g**, di‐*n*‐propylamine **2 h**, di‐*n*‐butylamine **2 i**, pyrrolidine **2 j**, piperidine **2 k**, morpholine **2 l**, furnished the corresponding sulfonamides in moderate yields (Table 2, entries 7–12). We further investigated the scope of substrates of anilines in this reaction, the results showed that poorly nucleophilic anilines **2 m**–**2 q** were utilized as the substrates and gave the sulfonamides **3 m**–**3 q** in moderate yields (Table 2, entries 13–17).

**Table 2:** Substrate scope of amines.^[a]^

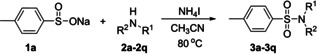			
Entry	Amines	Products	Yield^[b]^
1	**2 a**	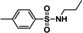	**3 a**, 74 %
2	**2 b**	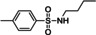	**3 b**, 71 %
3	**2 c**	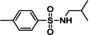	**3 c**, 70 %
4	**2 d**	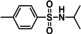	**3 d**, 60 %
5	**2 e**	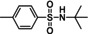	**3 e**, 45 %
6	**2 f**	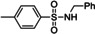	**3 f**, 75 %
7	**2 g**	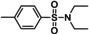	**3 g**, 82 %
8	**2 h**	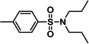	**3 h**, 65 %
9	**2 i**	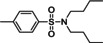	**3 i**, 60 %
10	**2 j**	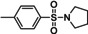	**3 j**, 78 %
11	**2 k**	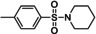	**3 k**, 70 %
12^[c]^	**2 l**	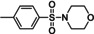	**3 l**, 60 %
13	**2 m**	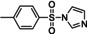	**3 m**, 63 %
14	**2 n**	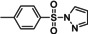	**3 n**, 60 %
15	**2 o**	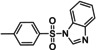	**3 o**, 66 %
16	**2 p**	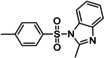	**3 p**, 62 %
17	**2 q**	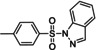	**3 q**, 60 %

[a] Reaction conditions: **1 a** (0.2 mmol), **2 a**–**2 q** (0.3 mmol), NH_4_I (0.2 mmol), CH_3_CN (2 mL), 80 °C, under air in Schlenk tube for 12 h. [b] isolated yield.

The scope of substrates with a variety of sodium sulfinates was also investigated in this reaction. As illustrated in Table 3, the sodium sulfinates substrates without substituent or with electron‐donating and electron‐withdrawing groups in the para‐position were found to be well‐tolerated, such as methyl (Table 3, entry 1), chloro (Table 3, entry 3) and nitro (Table 3, entry 4) substituted sulfonamides were obtained under the optimal reaction conditions. Especially, 2‐naphthyl sodium sulfinate **1 e** also reacted smoothly, and the corresponding sulfonamide **3 u** was acquired in 68 % yield (Table 3, entry 5).

**Table 3:** Substrate scope of sodium sulfinates.^[a]^

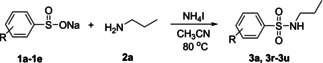			
Entry	Sodium sulfinate	Products	Yield^[b]^
1	**1 a**	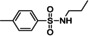	**3 a**, 75 %
2	**1 b**	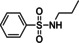	**3 r**, 77 %
3	**1 c**	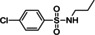	**3 s**, 73 %
4	**1 d**	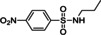	**3 t**, 73 %
5	**1 e**	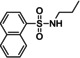	**3 u**, 68 %

[a] Reaction conditions: sodium sulfinates **1 a**–**1 e** (0.2 mmol), **2 a** (0.3 mmol), NH_4_I (0.2 mmol), CH_3_CN (2 mL), 80 °C, under air in Schlenk tube for 12 h. [b] isolated yield.

To aid understanding of the reaction mechanism, the free radical quencher TEMPO (2,2,6,6‐tetramethylpiperidine‐1‐oxyl) was added to the reaction system, only trace of **3 a** was detected, the result showed that that a free radical process was involved in the present reaction [Eq. 1].

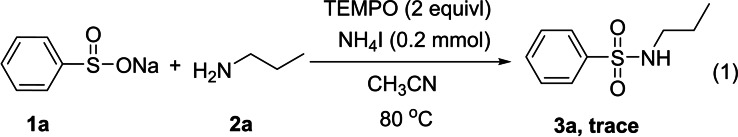




**Scheme 2:**
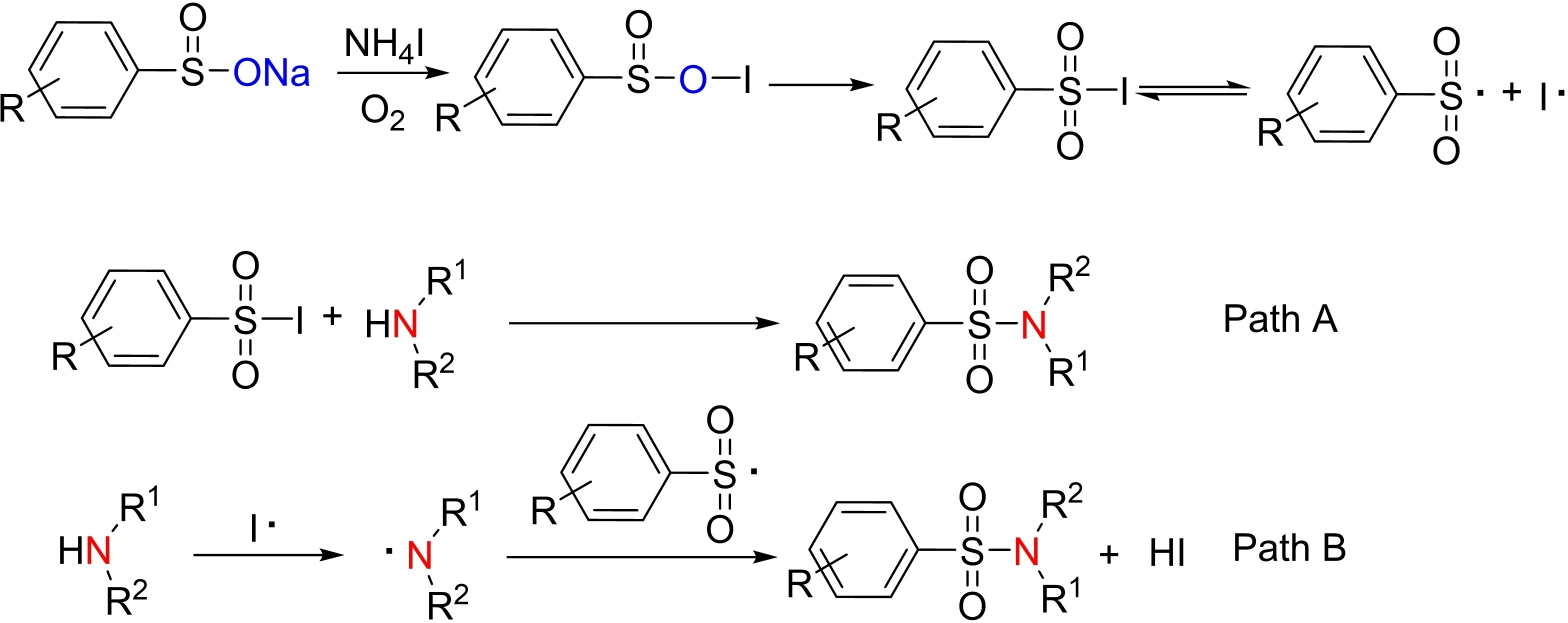
Plausible mechanism for the amination sodium sulfinates for the synthesis of sulfonamides.

Based on the above experimental results and referring to the previous reports,[^22^, ^24^] a possible reaction pathway was proposed and is shown in Scheme 2. The reaction between iodine (NH_4_I) and sodium sulfinate could lead to in situ formation of sulfonyl iodide intermediate, sulfonyl radical was produced by decomposition of sulfonyl iodide intermediate.^[24]^ Sulfonamide was formed via path A (displacement of iodide by the amine) or path B (radical substitution to S−N bond formation).

## Conclusion

In summary, we have developed a simple and effective method for the synthesis of sulfonamides through NH_4_I‐mediated sodium sulfinates and amines. This attractive and facile synthetic route has high functional groups tolerance to both aromatic and aliphatic amines and provides a variety of sulfonamide products in reasonable to excellent yields. As an alternative method to produce sulfonamides, the protocol has great applicability.

## Experimental Section

Unless otherwise noted, all reagents were obtained from commercial suppliers and used without further purification. Sodium sulfinates (0.2 mmol), amines (0.3 mmol), NH_4_I (0.2 mmol), were placed in a Schlenk tube (25 mL), and the mixture was stirred at 80 °C for 12 h, the reactions were monitored by GC and TLC. Then, the mixture was cooled to room temperature, washed with saturated NaCl solution. The crude product was extracted with ethyl acetate three times. The organic layer was dried over anhydrous Na_2_SO_4_, and concentrated under reduced pressure. The residue was purified by column chromatography on silica gel and eluted with petroleum to afford the analytically pure products.

## Conflict of interest

The authors declare no conflict of interest.

1

## Supporting information

As a service to our authors and readers, this journal provides supporting information supplied by the authors. Such materials are peer reviewed and may be re‐organized for online delivery, but are not copy‐edited or typeset. Technical support issues arising from supporting information (other than missing files) should be addressed to the authors.

Supporting Information

## Data Availability

Research data are not shared.
