# Supplementary material for: An Efficient Method for the Preparation of Sulfonamides from Sodium Sulfinates and Amines
Source: ChemistryOpen. 2022 Aug 25;11(8):e202200097. doi: 10.1002/open.202200097 (PMC9405518; doi:10.1002/open.202200097)
Supplement: Supplementary file 1 — Supporting Information [file OPEN-11-e202200097-s001.pdf]

ChemistryOpen

Supporting Information

An Efficient Method for the Preparation of Sulfonamides from Sodium Sulfinates and Amines

Haiying Tian, Ruiyan Li, Fang Guo, and Xiuling Chen*

Contents

1. General method...S2

2. General procedure……...S2

3. Characterization results of products **3a-3u**...S2-S6

4. References……..S6

5. ^{1}H and ^{13}C NMR spectra of compound **3a-3u**...S7-S27

General method

All reagents and solvents were used without further purification. The reactions were monitored by GC and GC-MS. The ^{1}H NMR and ^{13}C NMR spectra were recorded on a Brucker ADVANCE III spectrometer at 400 MHz and 100 MHz respectively. Thin layer chromatography (TLC) employed glass 0.25 mm silica gel plates. Flash chromatography columns were packed with 300-400 mesh silica gel in petroleum (bp. 60-90 ^{o}C). GC-MS result was recorded on GC-MS QP2010, and GC analysis was performed on GC 2010 plus. Sodium sulfinates and amines were purchased from Energy Chemical, Alfa, Aladdin.

2. General procedure

Sodium sulfinates (0.2 mmol), amines (0.3 mmol), were placed in a Schlenk tube (25 mL), and the mixture was stirred at 80 °C for 10 h, the reactions were monitored by GC and TLC. Then, the mixture was cooled to room temperature, washed with saturated NaCl solution. The crude product was extracted with ethyl acetate three times. The organic layer was dried over anhydrous Na_2SO_4, and concentrated under reduced pressure. The residue was purified by column chromatography on silica gel and eluted with petroleum to afford the analytically pure products.

3. Characterization results of products 3a-3u

4-Methyl-*N*-propylbenzenesulfonamide (**3a**)[1]

The title compound was prepared according to the general procedure and purified by column chromatography on silica gel and eluted with petroleum ether/ethyl acetate (10:1) to afford the desired product. Colorless liquid, isolated yield: 74%. ^{1}H NMR (400 MHz, $CDCl_3$): δ 7.76 (d, *J* = 8.4 Hz, 2H), 7.29 (d, *J* = 8.0 Hz, 2H), 5.19 (t, *J* = 5.6 Hz, 1H), 2.88 (q, *J* = 6.8 Hz, 2H), 2.42 (s, 3H), 1.43-1.52 (m, 2H), 0.85 (t, *J* = 7.4 Hz, 3H); ^{13}C NMR (100 MHz, CDCl3): δ 143.3, 137.0, 129.7, 127.1, 44.9, 22.8, 21.5, 11.1. MS (EI), m/z = 213.08.

N-Butyl-4-methylbenzenesulfonamide (**3b**)[1]

The title compound was prepared according to the general procedure and purified by column chromatography on silica gel and eluted with petroleum ether/ethyl acetate (10:1) to afford the desired product. Colorless liquid, isolated yield: 71%. ^{1}H NMR (400 MHz, $CDCl_3$): δ 7.75 (d, *J* = 8.4 Hz, 2H), 7.29 (d, *J* = 8.0 Hz, 2H), 4.72 (t, *J* = 6.0 Hz, 1H), 2.93 (q, *J* = 6.8 Hz, 2H), 2.43 (s, 3H), 1.40-1.47 (m, 2H), 1.24-1.33 (m, 2H), 0.84 (d, *J* = 7.4 Hz, 3H); ^{13}C NMR (100 MHz, $CDCl_3$): δ 143.3, 137.0, 129.7, 127.1, 42.9, 31.5, 21.5, 19.7, 13.5. MS (EI), m/z = 227.10.

N-Isobutyl-4-methylbenzenesulfonamide (**3c**)[2]

The title compound was prepared according to the general procedure and purified by column chromatography on silica gel and eluted with petroleum ether/ethyl acetate (10:1) to afford the desired product. Colorless liquid, isolated yield: 70%. ^{1}H NMR (400 MHz, $CDCl_3$): 7.77 (d, *J* = 8.4 Hz, 2H),

7.28 (d, J = 8.0 Hz, 2H), 5.46 (t, J = 6.4 Hz, 1H), 2.72 (t, J = 6.6 Hz, 2H), 2.42 (s, 3H), 1.43-1.52 (m, 2H), 0.84 (t, J = 6.8 Hz, 3H); ^{13}C NMR (100 MHz, $CDCl_3$): δ 143.1, 137.1, 129.6, 127.0, 50.5, 28.4, 21.4, 19.9. MS (EI), m/z = 227.10.

N-Isopropyl-4-methylbenzenesulfonamide (**3d**)[2]

The title compound was prepared according to the general procedure and purified by column chromatography on silica gel and eluted with petroleum ether/ethyl acetate (10:1) to afford the desired product. Colorless liquid, isolated yield: 60%. ^{1}H NMR (400 MHz, $CDCl_3$): 7.77 (d, J = 8.4 Hz, 2H), 7.28 (d, J = 8.0 Hz, 2H), 4.88 (d, J = 7.2 Hz, 1H), 3.39-3.47 (m, 1H), 2.42 (s, 3H), 1.06 (d, J = 6.8 Hz, 6H); ^{13}C NMR (100 MHz, $CDCl_3$): δ 143.2, 138.2, 129.6, 127.0, 46.0, 23.7, 21.5. MS (EI), m/z = 213.08.

N-(Tert-butyl)-4-methylbenzenesulfonamide (**3e**)[6]

The title compound was prepared according to the general procedure and purified by column chromatography on silica gel and eluted with petroleum ether/ethyl acetate (10:1) to afford the desired product. Colorless liquid, isolated yield: 45%. ^{1}H NMR (400 MHz, $CDCl_3$): 7.76 (d, J = 8.4 Hz, 2H), 7.26 (d, J = 8.4 Hz, 2H), 4.55 (s, 1H), 2.42 (s, 3H), 1.22 (s, 9H); ^{13}C NMR (100 MHz, CDCl3): δ 142.8, 140.5, 129.5, 127.0, 30.2, 21.5. MS (EI), m/z = 227.10.

N-Benzyl-4-methylbenzenesulfonamide (**3f**)[3]

The title compound was prepared according to the general procedure and purified by column chromatography on silica gel and eluted with petroleum ether/ethyl acetate (10:1) to afford the desired product. Colorless liquid, isolated yield: 75%. ^{1}H NMR (400 MHz, $CDCl_3$): δ 7.73 (d, J = 8.4 Hz, 2H), 7.23-7.29 (m, 5H), 7.19 (d, J = 8.4 Hz, 2H), 4.92 (t, J = 6.0 Hz, 1H), 4.09 (d, J = 6.0 Hz, 2H), 2.43 (s, 3H); ^{13}C NMR (100 MHz, $CDCl_3$): δ 1.43.5, 136.9, 136.4, 129.7, 128.7, 127.88, 128.86, 127.2, 47.3, 21.5. MS (EI), m/z = 261.08.

N,N-Diethyl-4-methylbenzenesulfonamide (**3g**)[1]

The title compound was prepared according to the general procedure and purified by column chromatography on silica gel and eluted with petroleum ether/ethyl acetate (10:1) to afford the desired product. Colorless liquid, isolated yield: 82%. ^{1}H NMR (400 MHz, $CDCl_3$): δ 7.77 (d, J = 8.4 Hz, 2H), 7.32 (d, J = 8.0 Hz, 2H), 3.28 (q, J = 4.8 Hz, 2H), 3.17 (q, J = 7.2 Hz, 2H), 2.45 (s, 3H), 0.83 (t, J = 7.4 Hz, 3H), 0.78 (t, J = 7.4 Hz, 3H); ^{13}C NMR (100 MHz, $CDCl_3$): δ 143.4, 137.1, 129.8, 127.2, 44.8, 21.5, 11.2. MS (EI), m/z = 227.09.

4-Methyl-*N,N*-dipropylbenzenesulfonamide (**3h**)[1]

The title compound was prepared according to the general procedure and purified by column chromatography on silica gel and eluted with petroleum ether/ethyl acetate (10:1) to afford the desired product. Colorless liquid, isolated yield: 65%. ^{1}H NMR (400 MHz, $CDCl_3$): δ 7.74 (d, J = 8.4 Hz, 2H), 7.27 (d, J = 7.2 Hz, 2H), 3.39 (t, J = 7.6 Hz, 2H), 3.31 (t, J = 7.2 Hz, 2H), 2.39 (s, 3H), 1.25-1.31 (m,

4H), 0.94 (t, J = 7.2 Hz, 3H), 0.87 (t, J = 7.4 Hz, 3H); ^{13}C NMR (100 MHz, $CDCl_3$): δ 158.7, 142.2, 129.2, 126.3, 52.3, 21.4, 19.6, 13.5. MS (EI), m/z = 255.13.

N,N-Dibutyl-4-methylbenzenesulfonamide (**3i**)[1]

The title compound was prepared according to the general procedure and purified by column chromatography on silica gel and eluted with petroleum ether/ethyl acetate = 10:1. Colorless liquid, isolated yield: 60%. ^{1}H NMR (400 MHz, $CDCl_3$): δ 7.78 (d, J = 8.4 Hz, 2H), 7.28 (d, J = 3.2 Hz, 2H), 3.41 (t, J = 7.6 Hz, 2H), 3.31 (t, J = 7.2 Hz, 2H), 2.42 (s, 3H), 1.49-1.62 (m, 4H), 1.23-1.37 (m, 4H), 0.96 (t, J = 7.2 Hz, 3H), 0.89 (t, J = 7.4 Hz, 3H); ^{13}C NMR (100 MHz, $CDCl_3$): δ 158.7, 142.2, 129.2, 126.3, 52.3, 30.6, 21.4, 19.6, 13.4. MS (EI), m/z = 285.16.

1-Tosylpyrrolidine (**3j**)[4]

The title compound was prepared according to the general procedure and purified by column chromatography on silica gel and eluted with petroleum ether/ethyl acetate (20:1) to afford the desired product. Colorless liquid, isolated yield: 78%. ^{1}H NMR (400 MHz, $CDCl_3$): δ 7.70 (d, J = 8.4 Hz, 2H), 7.32 (d, J = 8.4 Hz, 2H), 3.22 (t, J = 6.8 Hz, 4H), 2.43 (s, 3H), 1.72-1.76 (m, 4H); ^{13}C NMR (100 MHz, $CDCl_3$): δ 143.4, 133.7, 129.6, 127.5, 47.9, 25.2, 21.5. MS (EI), m/z = 225.08.

1-Tosylpiperidine (**3k**)[4]

The title compound was prepared according to the general procedure and purified by column chromatography on silica gel and eluted with petroleum ether/ethyl acetate (20:1) to afford the desired product. Colorless liquid, isolated yield: 70%. ^{1}H NMR (400 MHz, $CDCl_3$): δ 7.65 (d, J = 8.4 Hz, 2H), 7.31 (d, J = 8.0 Hz, 2H), 2.97 (t, J = 5.6 Hz, 4H), 2.43 (s, 3H), 1.63 (t, J = 5.6 Hz, 4H), 1.28-1.44 (m, 2H); ^{13}C NMR (100 MHz, $CDCl_3$): δ 143.3, 133.3, 129.5, 127.7, 46.9, 25.2, 23.5, 21.5. MS (EI), m/z = 239.10.

4-Tosylmorpholine (**3l**)[4]

The title compound was prepared according to the general procedure and purified by column chromatography on silica gel and eluted with petroleum ether/ethyl acetate (10:1) to afford the desired product. Colorless liquid, isolated yield: 60%. ^{1}H NMR (400 MHz, $CDCl_3$): δ 7.70 (d, J = 8.4 Hz, 2H), 7.32 (d, J = 8.4 Hz, 2H), 3.22 (t, J = 6.8 Hz, 4H), 2.43 (s, 3H), 1.72-1.76 (m, 4H); ^{13}C NMR (100 MHz, $CDCl_3$): δ 143.4, 133.77, 129.6, 127.5, 47.9, 25.2, 21.5. MS (EI), m/z = 241.08.

1-Tosyl-1H-imidazole (**3m**)[6]

The title compound was prepared according to the general procedure and purified by column chromatography on silica gel and eluted with petroleum ether/ethyl acetate (20:1) to afford the desired product. White solid, isolated yield: 63%. ^{1}H NMR (400 MHz, $CDCl_3$): δ 8.02 (s, 1H), 7.82 (d, J = 8.4 Hz, 2H), 7.36 (d, J = 8.0 Hz, 2H), 7.29 (s, 1H), 7.09 (s, 1H), 2.45 (s, 3H); ^{13}C NMR (100 MHz, $CDCl_3$): δ 146.3, 136.7, 134.9, 131.4, 130.4, 127.4, 117.4, 21.7. MS (EI), m/z =222.05.

1-Tosyl-1H-pyrazole (**3n**)[6]

The title compound was prepared according to the general procedure and purified by column chromatography on silica gel and eluted with petroleum ether/ethyl acetate (20:1) to afford the desired product. White solid, isolated yield: 60%. ^{1}H NMR (400 MHz, $CDCl_3$): δ 8.09 (d, *J* = 8.4 Hz, 1H), 7.87 (d, *J* = 8.0 Hz, 2H), 7.09 (s, 1H), 7.31 (d, *J* = 8.0 Hz, 2H), 6.38 (t, *J* = 0.8 Hz, 1H), 2.40 (s, 3H); ^{13}C NMR (100 MHz, $CDCl_3$): δ 145.9, 145.2, 134.1, 131.1, 130.1, 128.1, 108.7, 21.7. MS (EI), m/z = 222.05.

1-Tosyl-1H-benzo[d]imidazole (**3o**)

The title compound was prepared according to the general procedure and purified by column chromatography on silica gel and eluted with petroleum ether/ethyl acetate (20:1) to afford the desired product. White solid, isolated yield: 66%. ^{1}H NMR (400 MHz, $CDCl_3$): δ 8.39 (s, 1H), 7.86 (t, *J* = 7.0 Hz, 3H), 7.53 (s, 1H), 7.32-7.40 (m, 2H), 7.27 (d, *J* = 8.4 Hz, 2H), 2.36 (s, 3H); ^{13}C NMR (100 MHz, $CDCl_3$): δ 146.2, 144.0, 141.3, 134.6, 130.3, 127.2, 125.6, 124.8, 121.1, 112.5, 21.6. HRMS (EI): calcd for $C_{14}H_{12}N_2O_2S$: 272.0619; found: 272.0633.

2-Methyl-1-tosyl-1H-benzo[d]imidazole (**3p**)[7]

The title compound was prepared according to the general procedure and purified by column chromatography on silica gel and eluted with petroleum ether/ethyl acetate (20:1) to afford the desired product. White solid, isolated yield: 62%. ^{1}H NMR (400 MHz, $CDCl_3$): δ 8.01 (d, *J* = 6.8 Hz, 1H), 7.79 (d, *J* = 8.4 Hz, 2H), 7.61 (d, *J* = 7.2 Hz, 1H), 7.31-7.34 (m, 2H), 2.25 (d, *J* = 8.0 Hz, 2H), 2.81 (s, 3H), 2.36 (s, 3H); ^{13}C NMR (100 MHz, $CDCl_3$): δ 151.4, 146.0, 141.9, 135.4, 133.2, 130.3, 126.8, 124.7, 124.6, 119.7, 113.4, 21.6, 16.9. HRMS (EI): calcd for $C_{15}H_{14}N_2O_2S$: 286.0776; found: 286.0762.

1-Tosyl-1H-indazole (**3q**)

The title compound was prepared according to the general procedure and purified by column chromatography on silica gel and eluted with petroleum ether/ethyl acetate (10:1) to afford the desired product. White solid, isolated yield: 60%. ^{1}H NMR (400 MHz, $CDCl_3$): δ 8.30 (s, 1H), 7.78 (t, *J* = 7.0 Hz, 3H), 7.67 (s, 1H), 7.23-7.31 (m, 2H), 7.19 (d, *J* = 8.4 Hz, 2H), 2.27 (s, 3H); ^{13}C NMR (100 MHz, $CDCl_3$): δ 146.3, 144.1, 141.3, 134.7, 130.4, 127.3, 125.6, 124.8, 121.1, 112.6, 21.7. HRMS (EI): calcd for $C_{14}H_{12}N_2O_2S$: 272.0619; found: 272.0633.

N-propylbenzenesulfonamide (**3r**)[2]

The title compound was prepared according to the general procedure and purified by column chromatography on silica gel and eluted with petroleum ether/ethyl acetate (10:1) to afford the desired product. White solid, isolated yield: 77%. ^{1}H NMR (400 MHz, $CDCl_3$): δ 7.88 (d, *J* = 7.2 Hz, 2H), 7.58 (t, *J* = 8.0 Hz, 1H), 7.19 (d, *J* = 7.8 Hz, 2H), 2.95 (q, *J* = 6.6 Hz, 2H), 1.47-1.57 (m, 2H), 0.88 (t, *J* = 7.4 Hz, 3H); ^{13}C NMR (100 MHz, $CDCl_3$): δ 134.2, 133.9, 128.9, 127.8, 45.0, 23.0, 11.1. MS (EI), m/z =199.26.

4-Chloro-N-propylbenzenesulfonamide (**3s**)[7]

The title compound was prepared according to the general procedure and purified by column chromatography on silica gel and eluted with petroleum ether/ethyl acetate (20:1) to afford the desired product. Colorless liquid, isolated yield: 73%. ^{1}H NMR (400 MHz, $CDCl_3$): δ 7.82 (d, *J* = 8.8 Hz, 2H),7.47 (d, *J* = 8.8 Hz, 2H), 4.77 (t, *J* = 6.0 Hz, 1H), 2.92 (q, *J* = 6.8 Hz, 2H), 1.42-1.53 (m, 2H), 0.86 (t, *J* = 7.4 Hz, 3H); ^{13}C NMR (100 MHz, $CDCl_3$): δ 139.0, 138.6, 129.4, 128.5, 44.9, 22.9, 11.0. MS (EI), m/z = 233.03.

4-Nitro-*N*-propylbenzenesulfonamide (**3t**)[7]

The title compound was prepared according to the general procedure and purified by column chromatography on silica gel and eluted with petroleum ether/ethyl acetate (20:1) to afford the desired product. Colorless liquid, isolated yield: 73%. ^{1}H NMR (400 MHz, $CDCl_3$): δ 7.74 (d, *J* = 8.8 Hz, 2H),7.42 (d, *J* = 8.8 Hz, 2H), 4.71 (t, *J* = 6.0 Hz, 1H), 2.86 (q, *J* = 6.8 Hz, 2H), 1.36-1.47 (m, 2H), 0.80 (t, *J* = 7.4 Hz, 3H); ^{13}C NMR (100 MHz, $CDCl_3$): δ 139.1, 138.6, 129.4, 128.5, 45.0, 22.9, 11.1. MS (EI), m/z =244.05.

N-Propylnaphthalene-1-sulfonamide (**3u**)[7]

The title compound was prepared according to the general procedure and purified by column chromatography on silica gel and eluted with petroleum ether/ethyl acetate (10:1) to afford the desired product. Colorless liquid, isolated yield: 68%. ^{1}H NMR (400 MHz, $CDCl_3$): δ 8.45 (d, *J* = 1.2 Hz, 1H),7.93 (d, *J* = 8.4 Hz, 2H), 7.86 (d, *J* = 1.6 Hz, 2H), 7.58-7.86 (m, 2H), 5.25 (t, *J* = 6.0 Hz, 1H), 2.92 (q, *J* = 8.4 Hz, 2H), 1.43-1.52 (m, 2H), 0.83 (t, *J* = 7.4 Hz, 3H); ^{13}C NMR (100 MHz, $CDCl_3$): δ 136.9, 134.8, 132.2, 129.5, 129.2, 128.7, 128.4, 127.9, 127.5, 122.4, 45.1, 23.0, 11.1. MS (EI), m/z = 249.08.

References

1. S. Y. Chow, M. Y. Stevens, L. R. S. Odell, *J. Org. Chem*., **2016**, *81*, 2681.
2. O. Chantarasriwong, D. Ok Jang, W. Chavasiri, *Tetrahedron Lett*., **2006**, *47*, 7489.
3. M. Harmata, P. Zheng, C. Huang, M. G. Gomes, W. Jing, K. Ranyanil, G. Balan and N. L. Calkins, *J. Org. Chem*., **2007**, *72*, 683.
4. R. Sridhar, B. Srinivas, V. P. Kumar, M. Narender and K. R. Rao, *Adv. Synth. Catal*., **2007**, *349*, 1873.
5. S. J. Gharpure, J. V. K. Prasad, *J. Org. Chem*., **2011**, *76*, 10325.
6. S. Yotphan, L. Sumunnee, D. Beukeaw, C. Buathongjan, V. Reutrakul, *Org. Biomol. Chem*., 2016, *14*, 590.
7. D. A. Powell, H. Fan, *J. Org. Chem*., **2010**, *75*, 8, 2726.

^{1}H and ^{13}C NMR spectra of compound **3a- 3u**

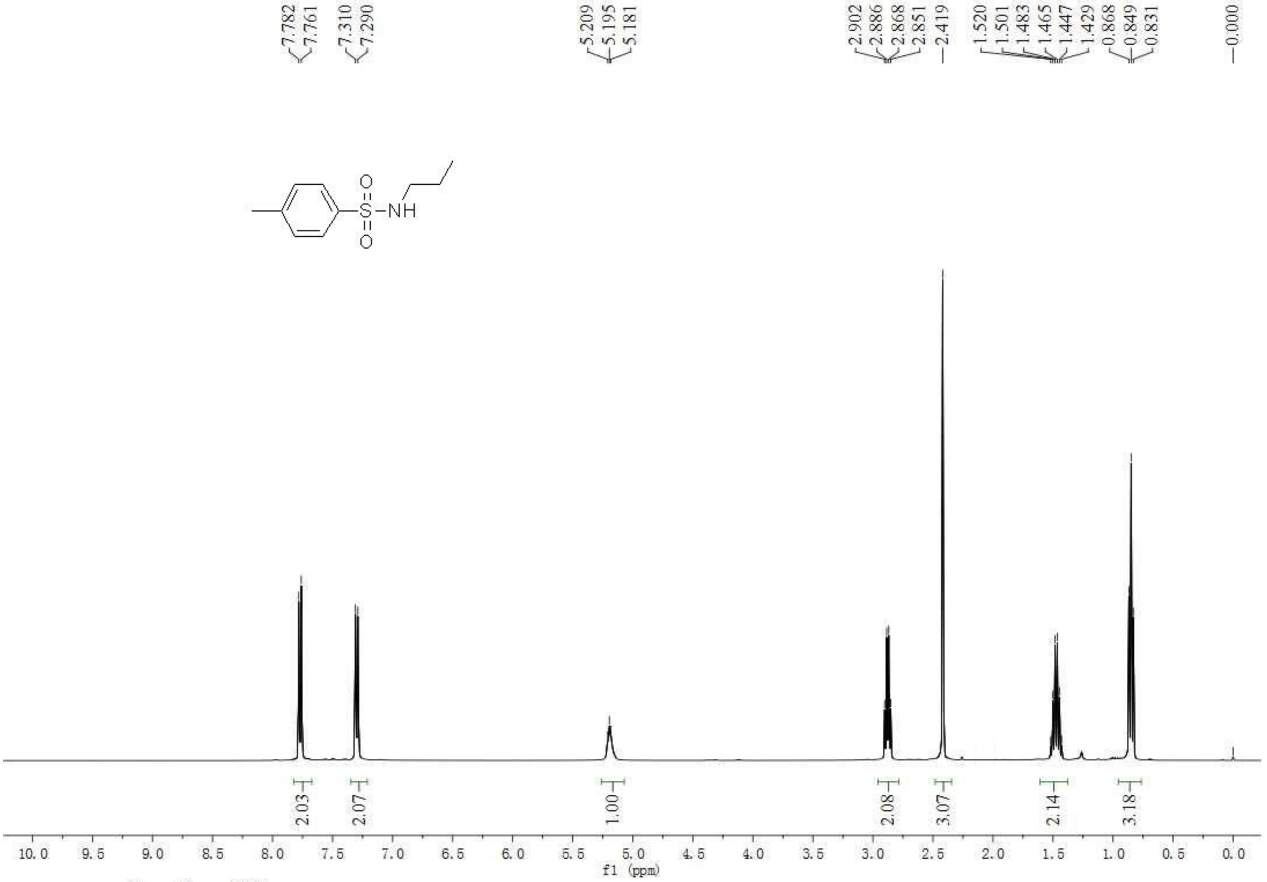

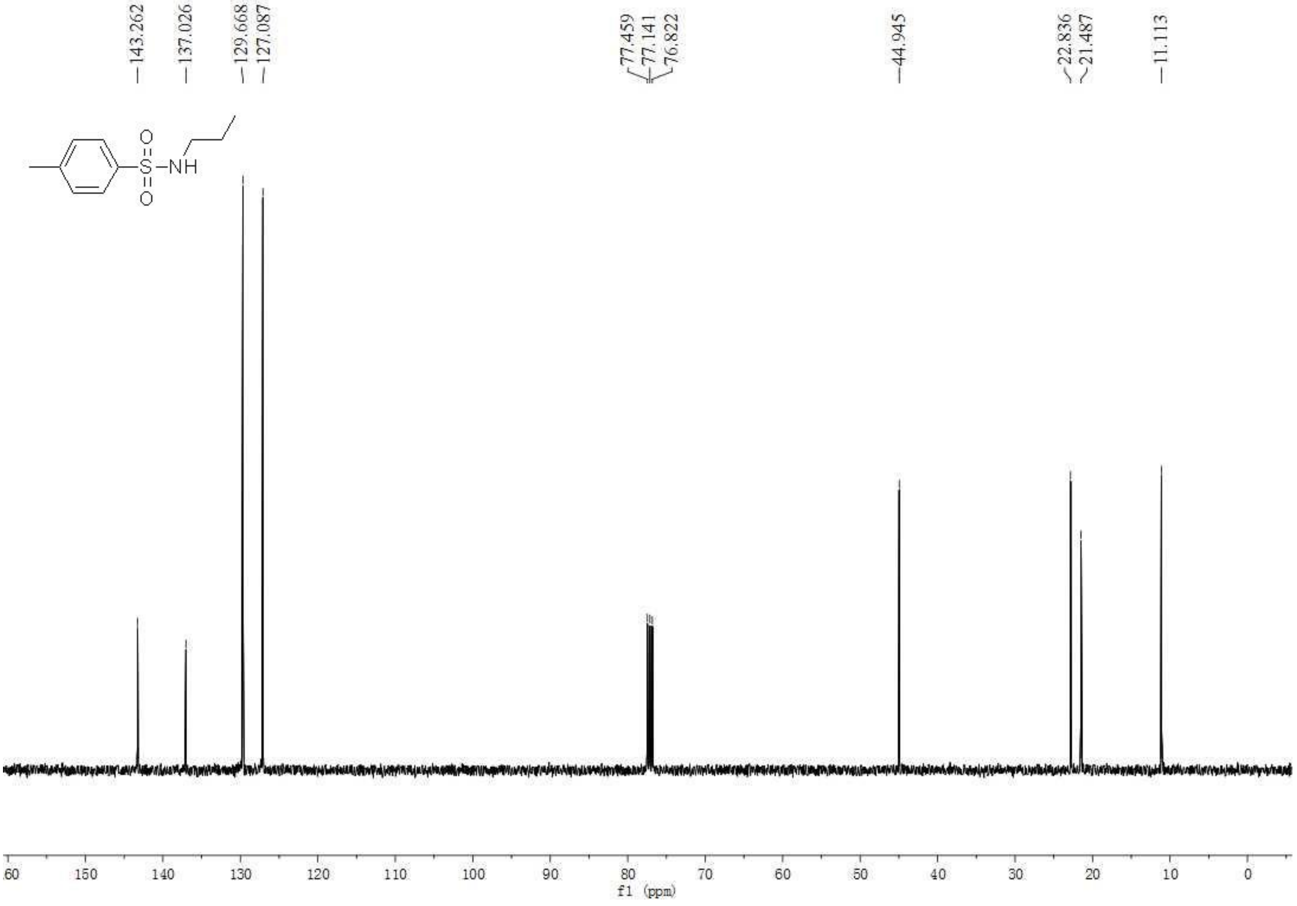

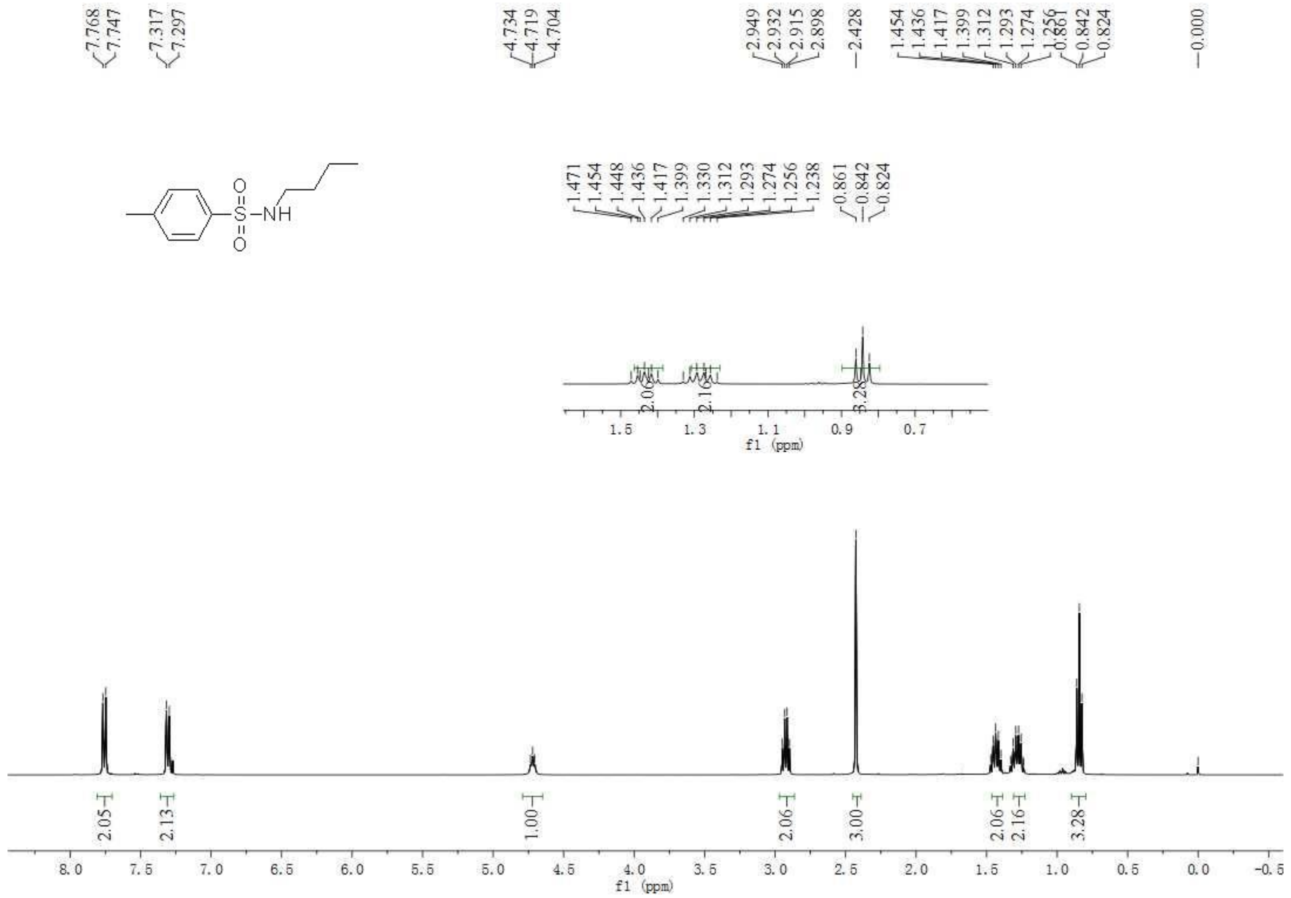
7.768
7.747
7.317
7.297
4.734
4.719
4.704
2.949
2.932
2.915
2.898
2.428
1.454
1.436
1.417
1.399
1.312
1.293
1.274
1.256
0.861
0.842
0.824
0.000
1.471
1.454
1.448
1.436
1.417
1.399
1.330
1.312
1.293
1.274
1.256
1.238
0.861
0.842
0.824
f1 (ppm)
2.05
2.13
1.00
2.06
3.00
2.06
2.16
3.28
f1 (ppm)

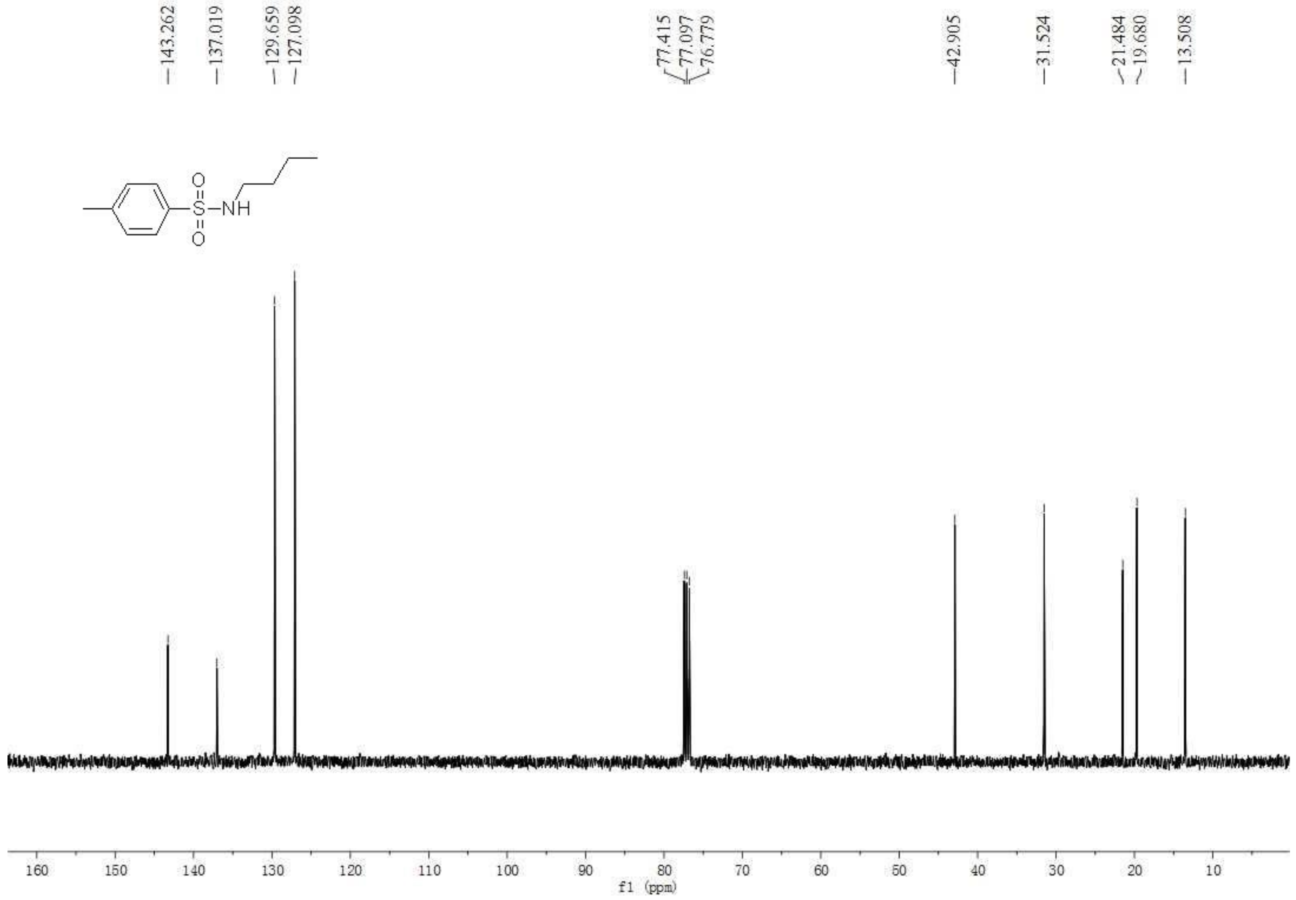
143.262
137.019
129.659
127.098
77.415
77.097
76.779
42.905
31.524
21.484
19.680
13.508
f1 (ppm)

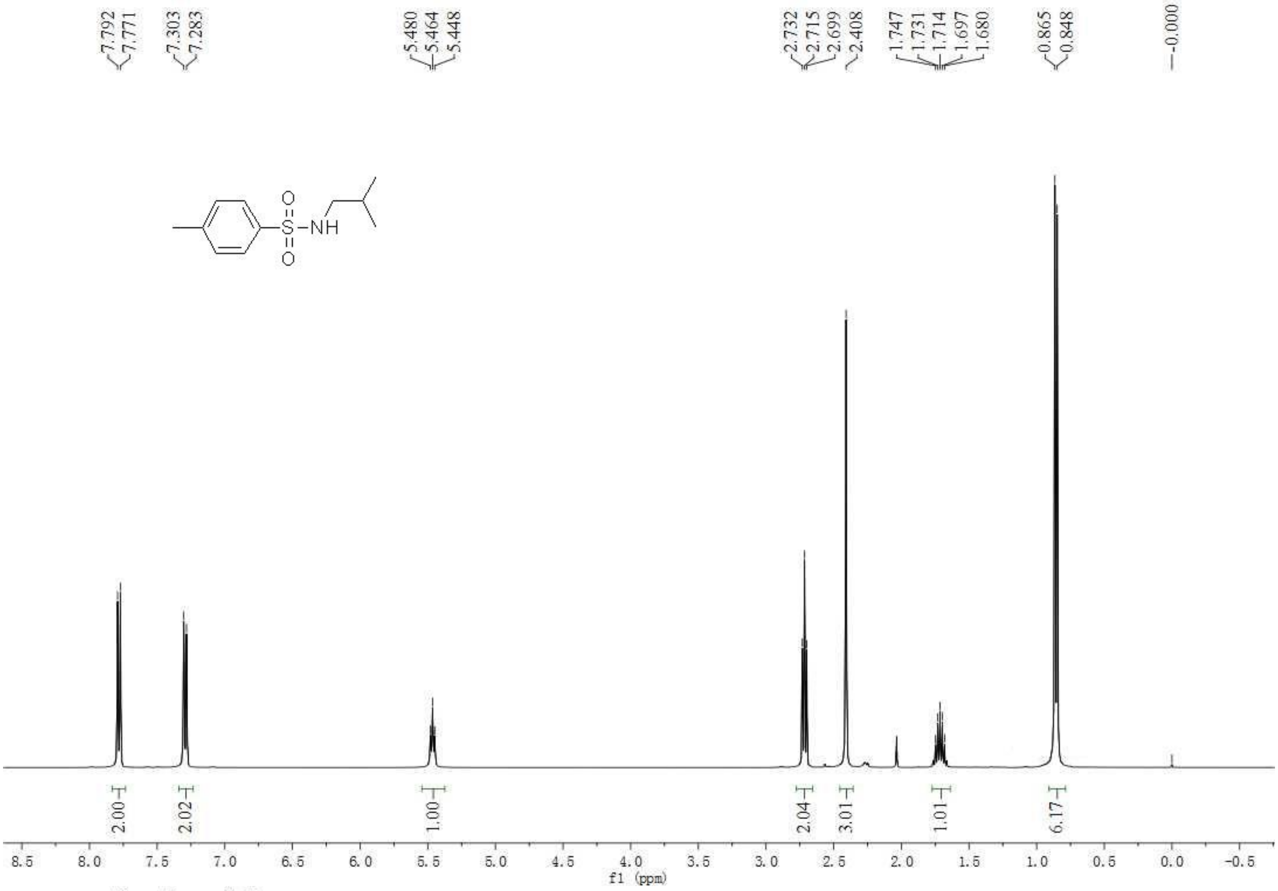

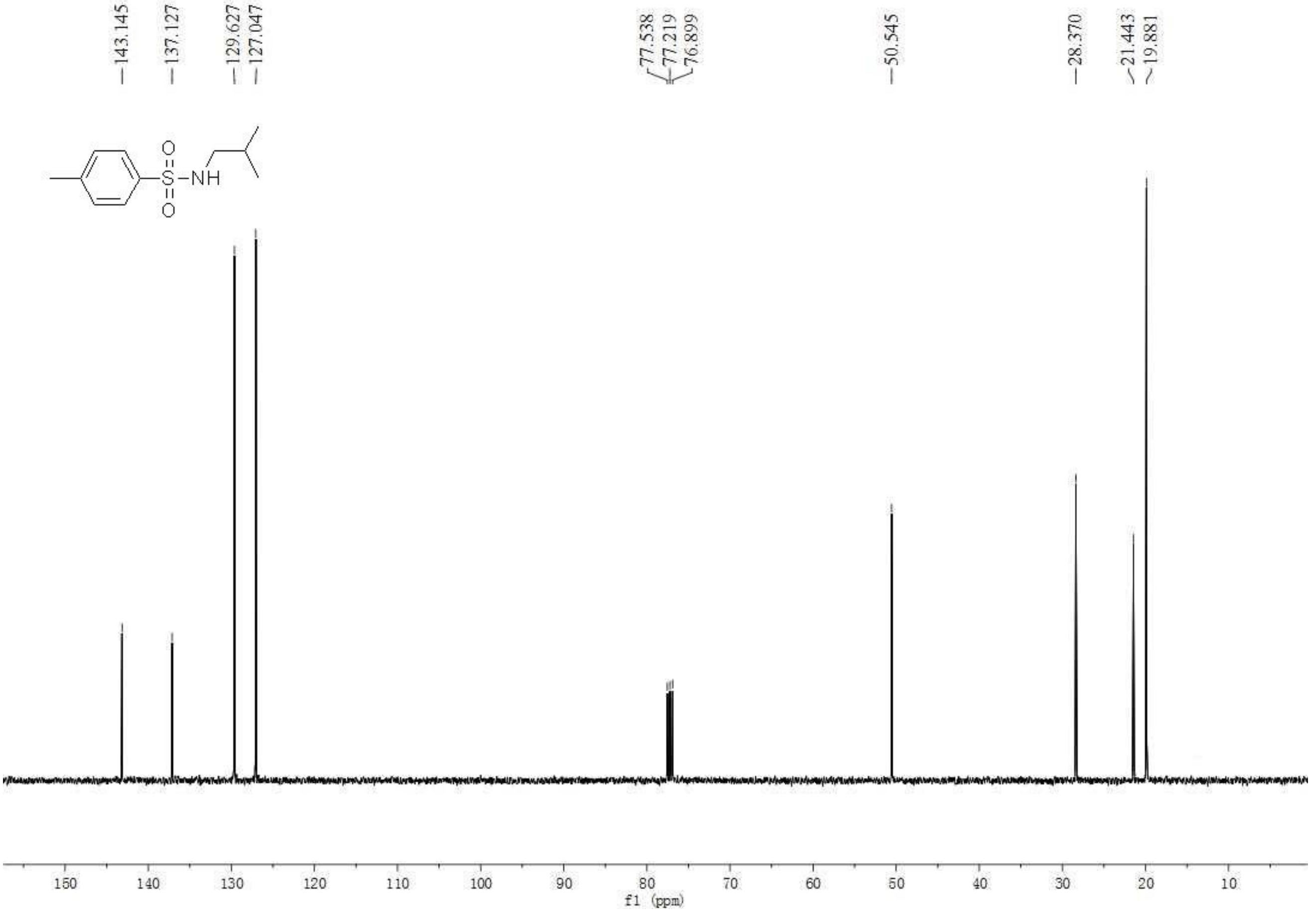

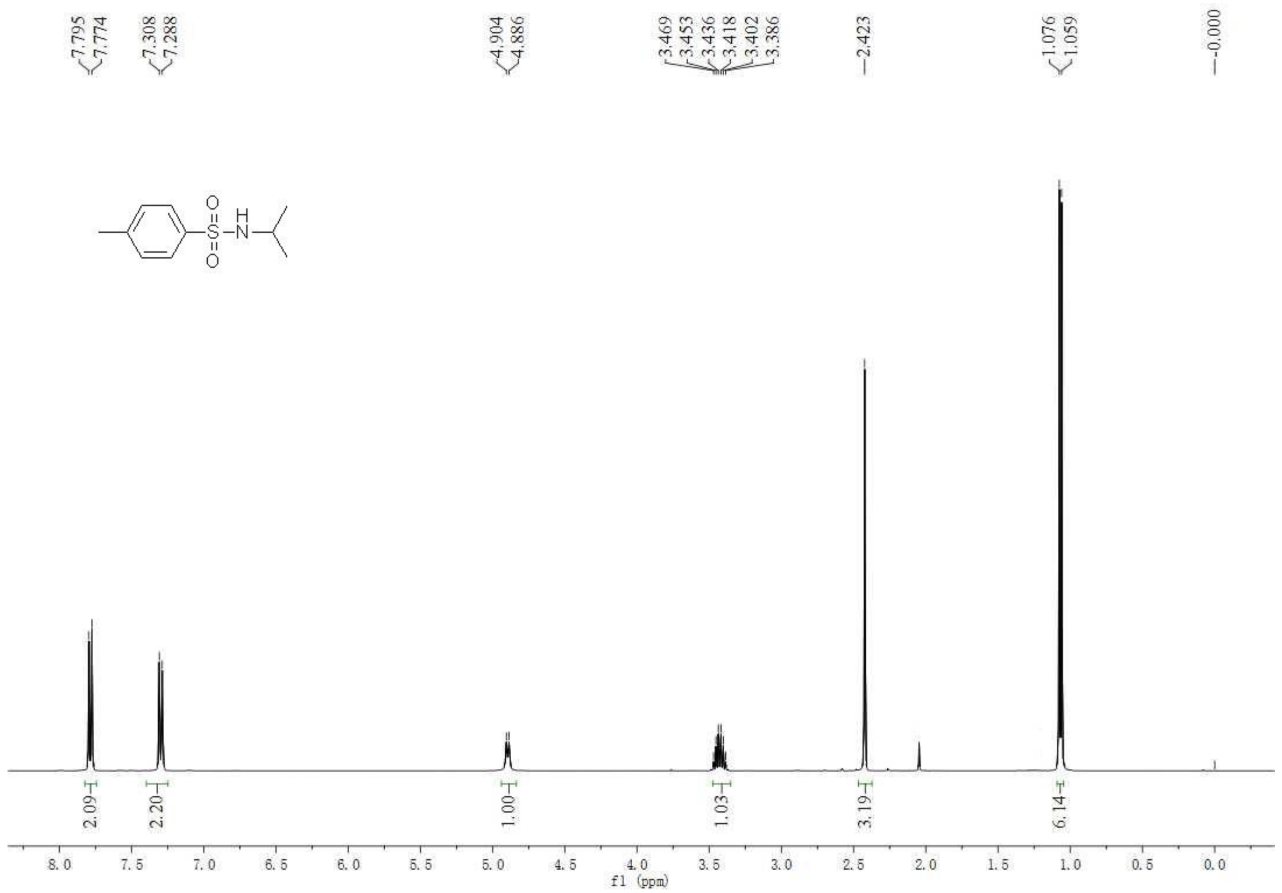
7.795
7.774
7.308
7.288
4.904
4.886
3.469
3.453
3.436
3.418
3.402
3.386
2.423
1.076
1.059
-0.000
2.09
2.20
1.00
1.03
3.19
6.14
8.0 7.5 7.0 6.5 6.0 5.5 5.0 4.5 4.0 3.5 3.0 2.5 2.0 1.5 1.0 0.5 0.0
f1 (ppm)

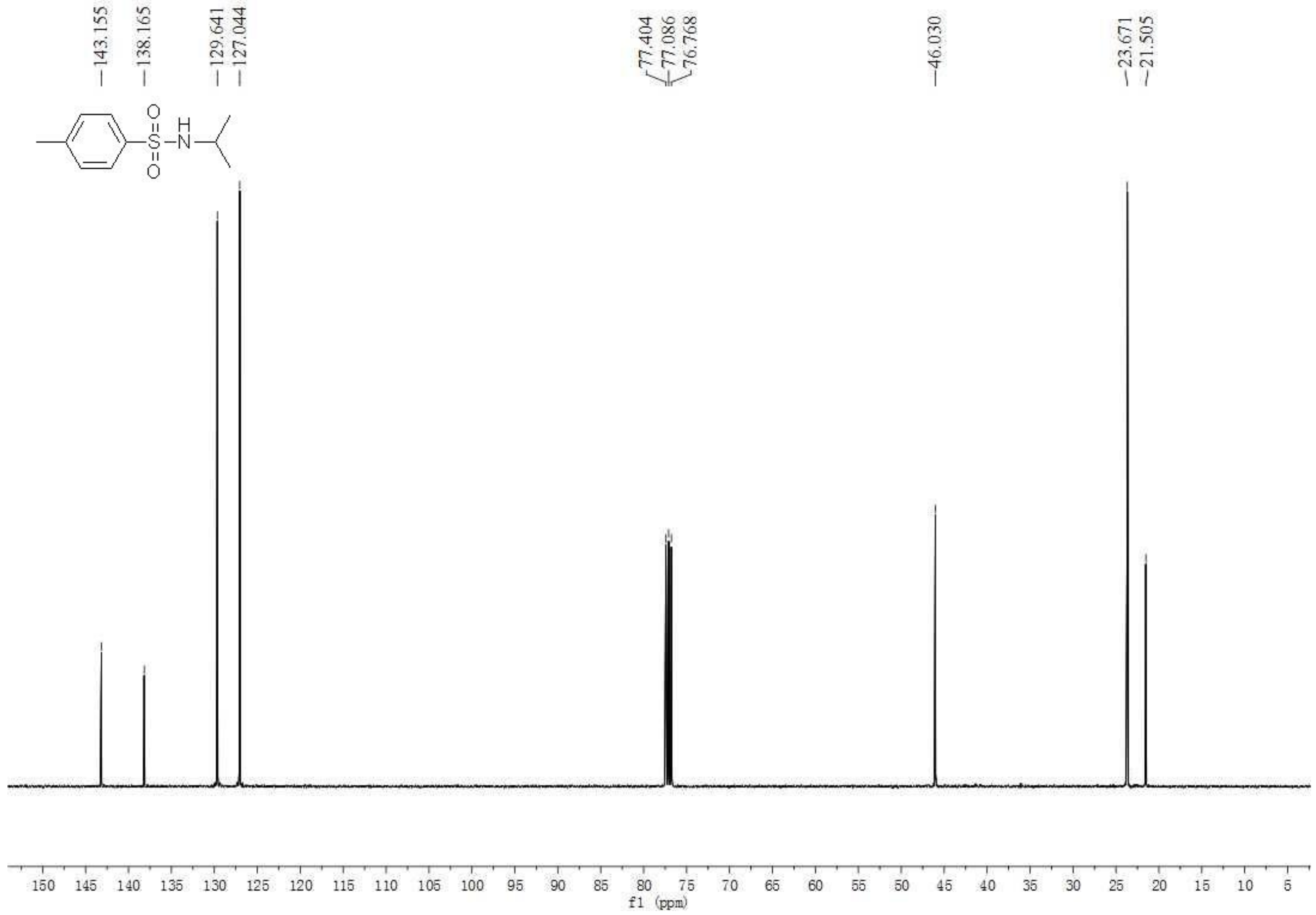
143.155
138.165
129.641
127.044
77.404
77.086
76.768
46.030
23.671
21.505
150 145 140 135 130 125 120 115 110 105 100 95 90 85 80 75 70 65 60 55 50 45 40 35 30 25 20 15 10 5
f1 (ppm)

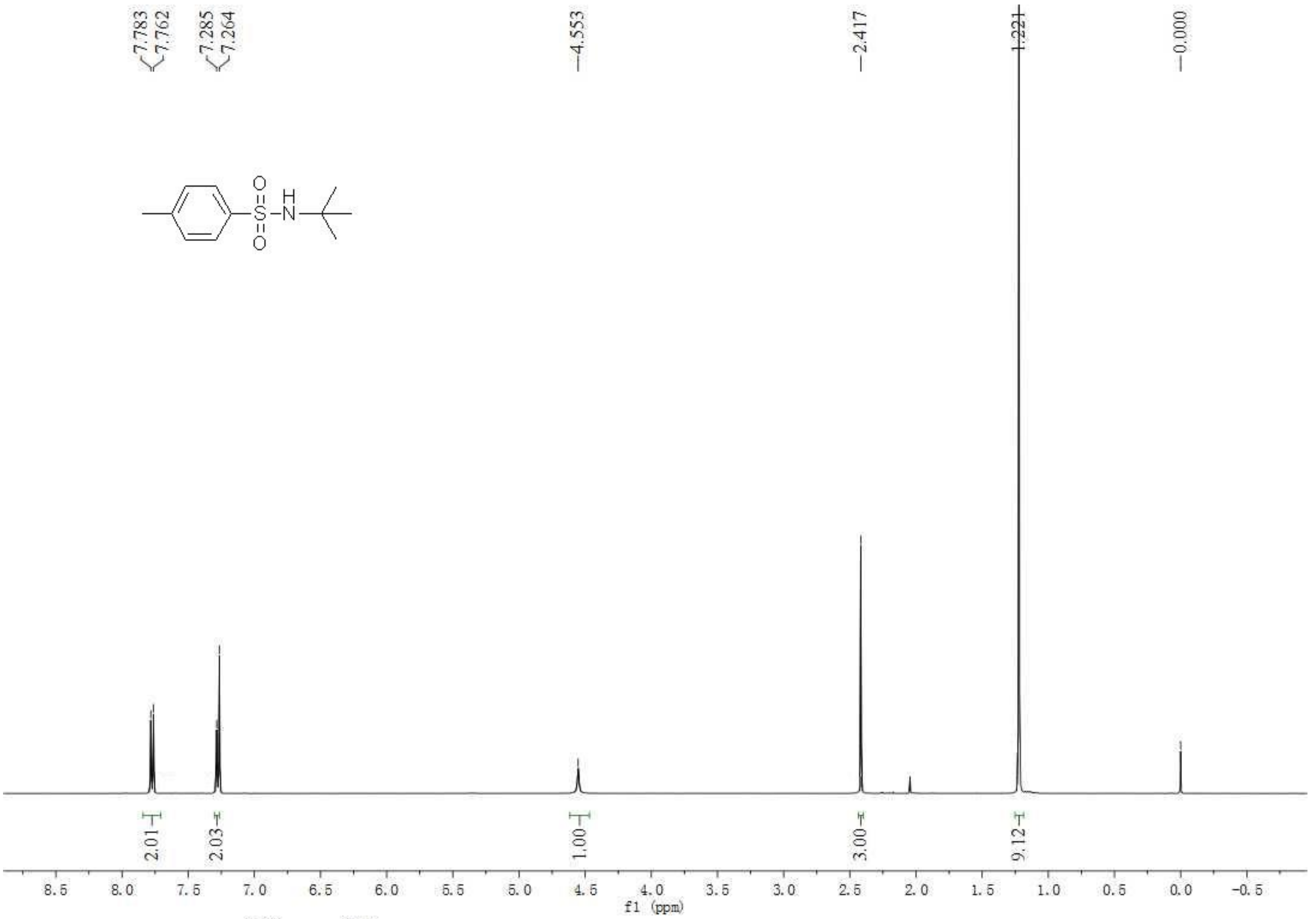
7.783
7.762
7.285
7.264
4.553
2.417
1.221
0.000
2.01
2.03
1.00
3.00
9.12
f1 (ppm)

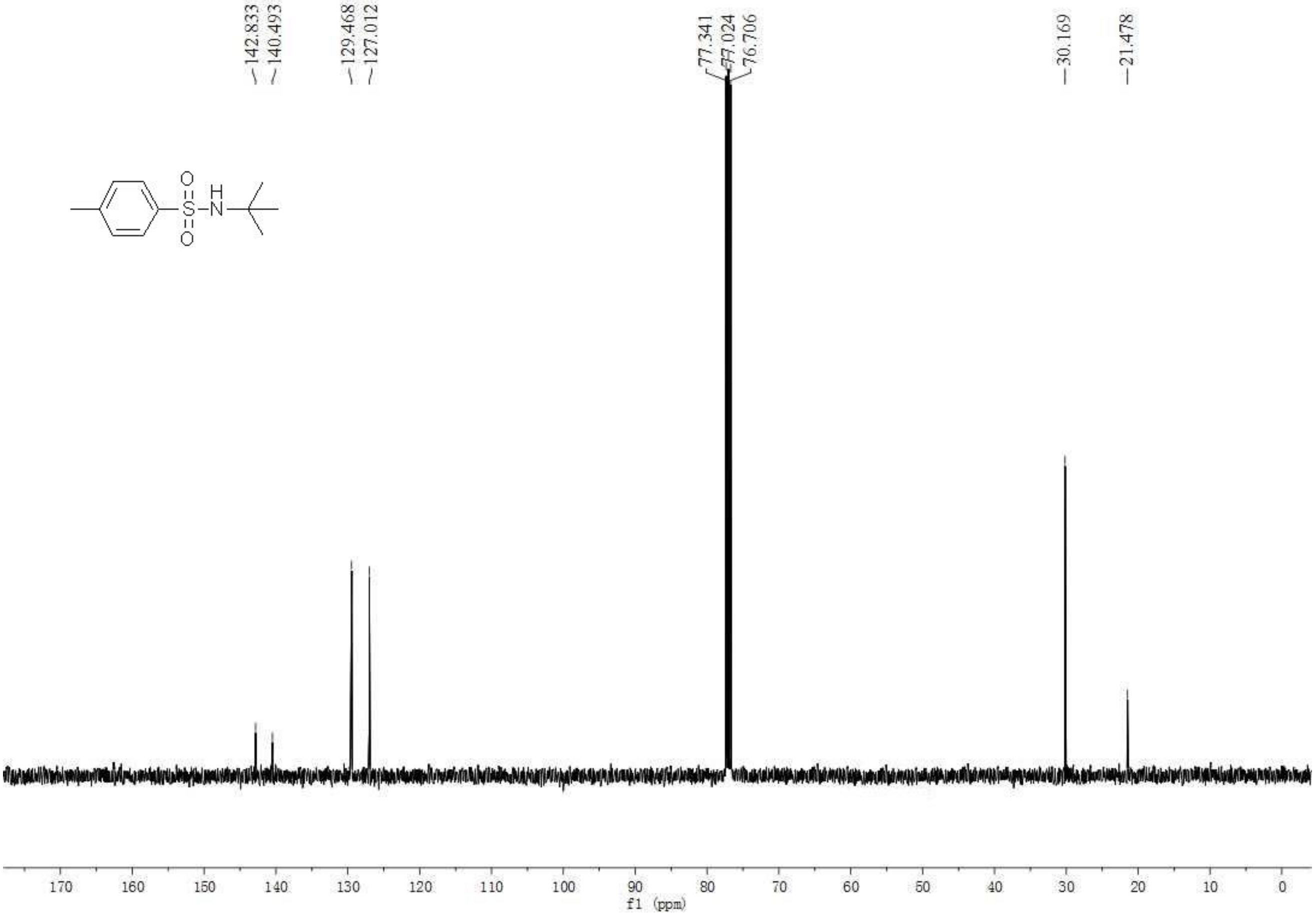
142.833
140.493
129.468
127.012
77.341
77.024
76.706
30.169
21.478
f1 (ppm)

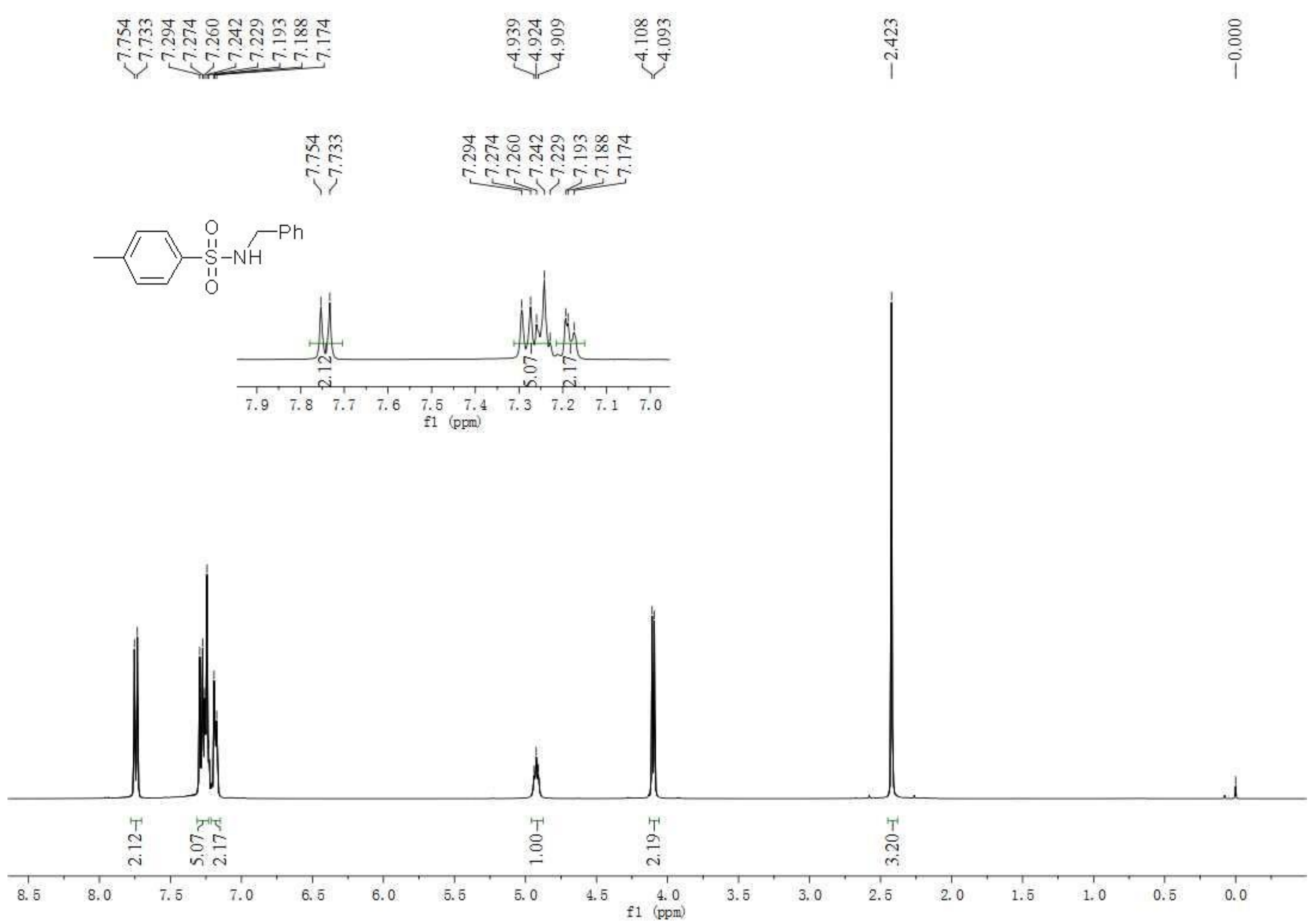
7.754
7.733
7.294
7.274
7.260
7.242
7.229
7.193
7.188
7.174
4.939
4.924
4.909
4.108
4.093
2.423
0.000
Ph
NH
S
O
2.12
5.07
2.17
1.00
2.19
3.20
f1 (ppm)

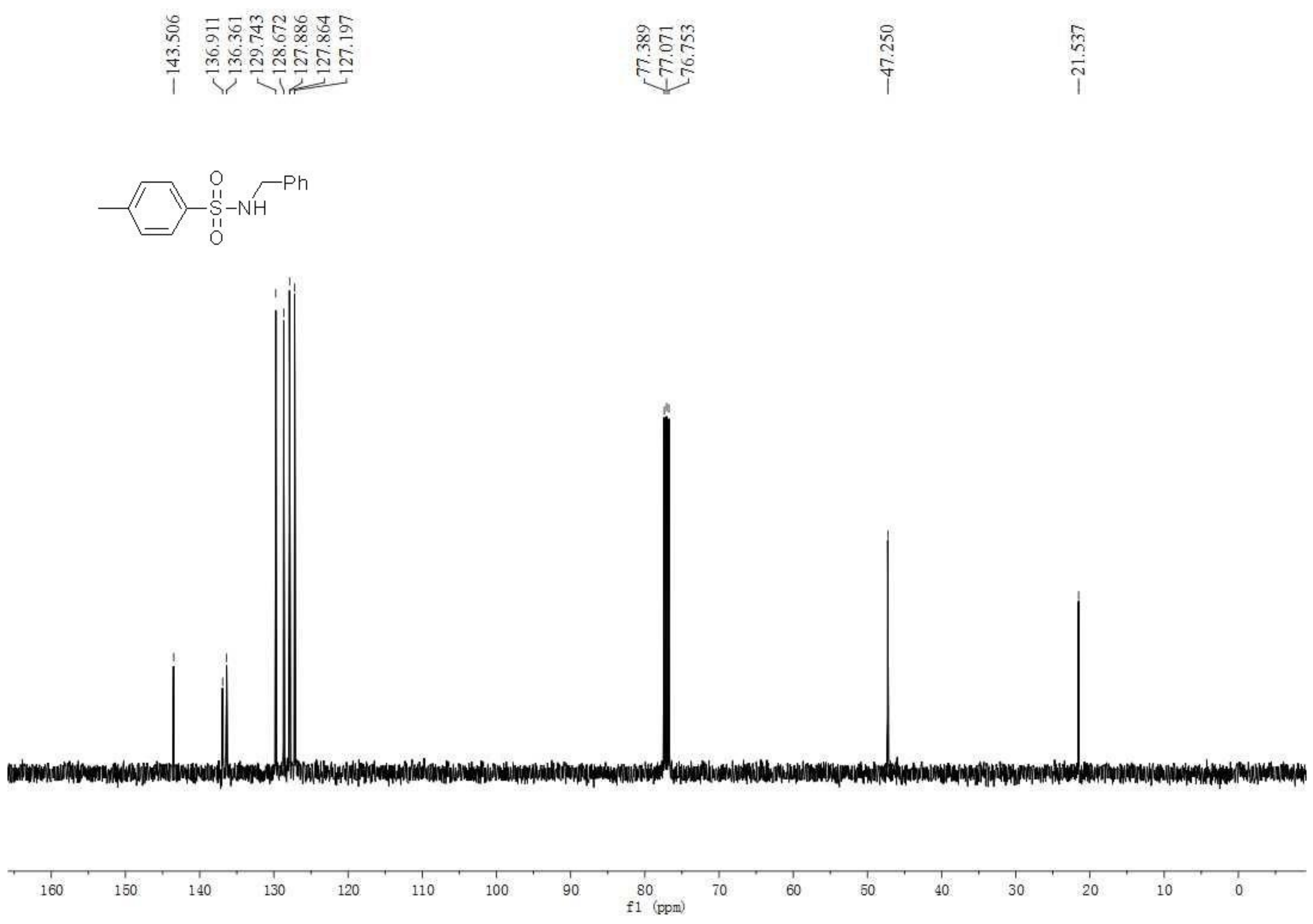
143.506
136.911
136.361
129.743
128.672
127.886
127.864
127.197
77.389
77.071
76.753
47.250
21.537
Ph
NH
S
O
f1 (ppm)

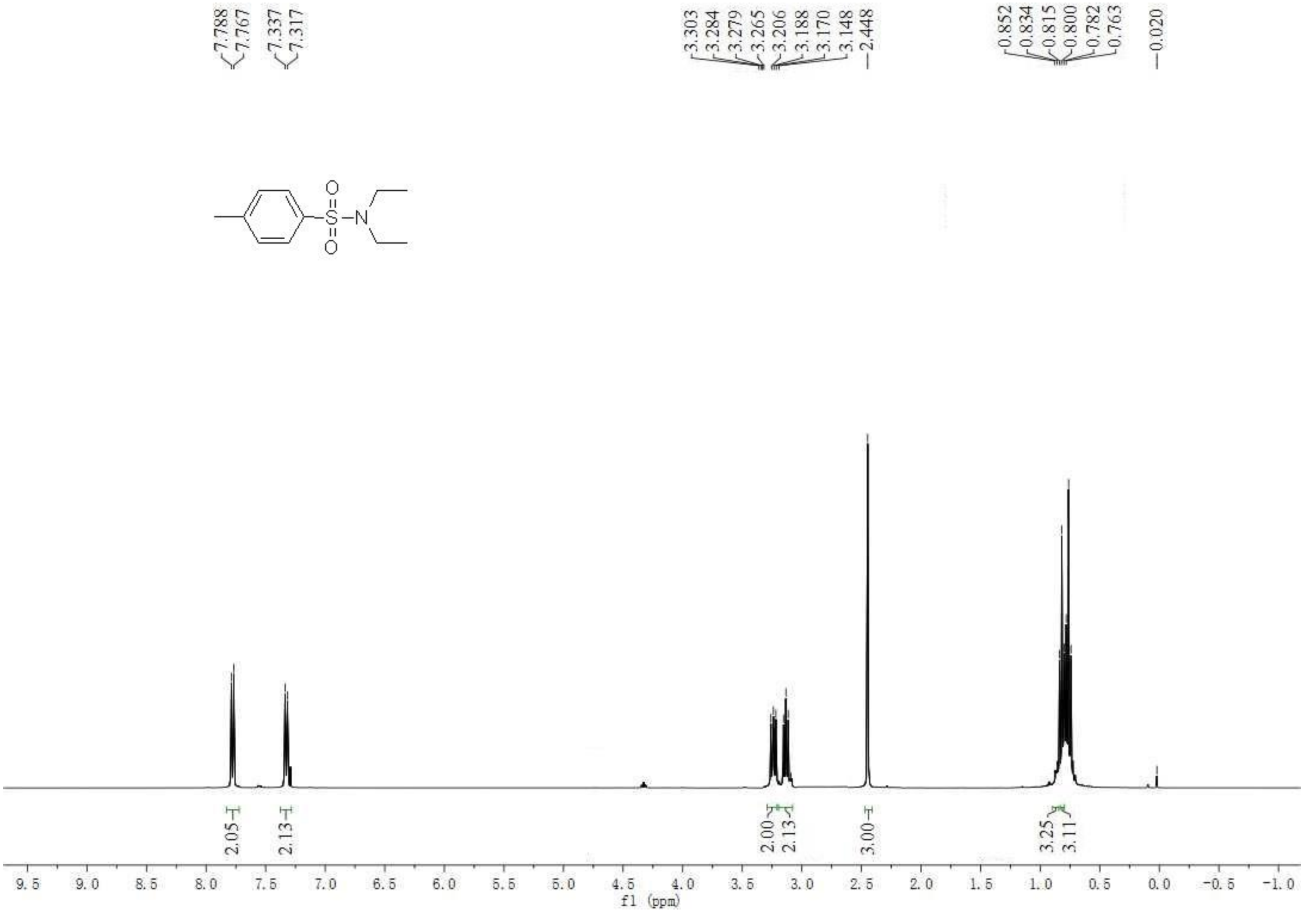
7.788
7.767
7.337
7.317
3.303
3.284
3.279
3.265
3.206
3.188
3.170
3.148
2.448
0.852
0.834
0.815
0.800
0.782
0.763
0.020
2.05
2.13
2.00
2.13
3.00
3.25
3.11
f1 (ppm)

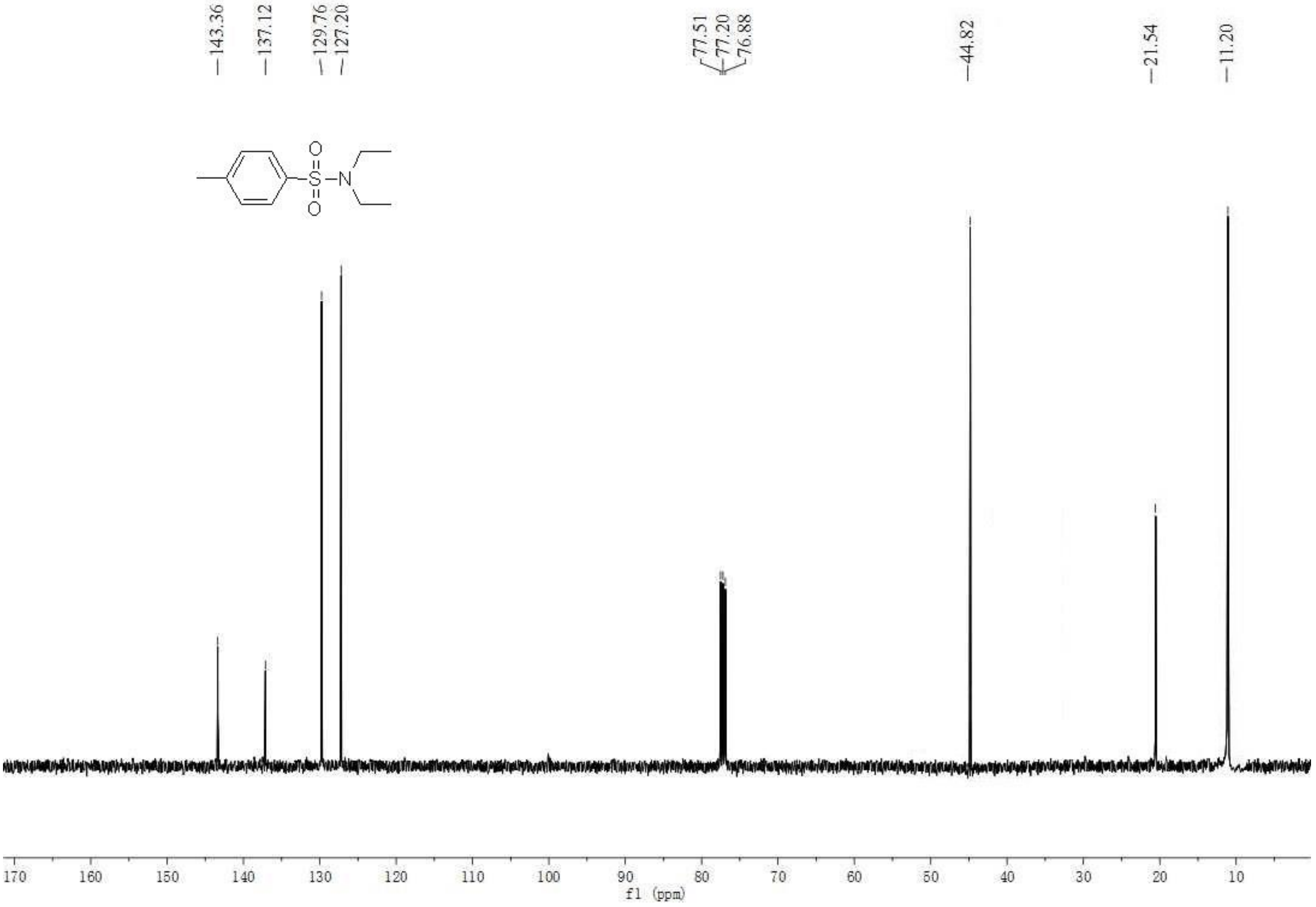
143.36
137.12
129.76
127.20
77.51
77.20
76.88
44.82
21.54
11.20
f1 (ppm)

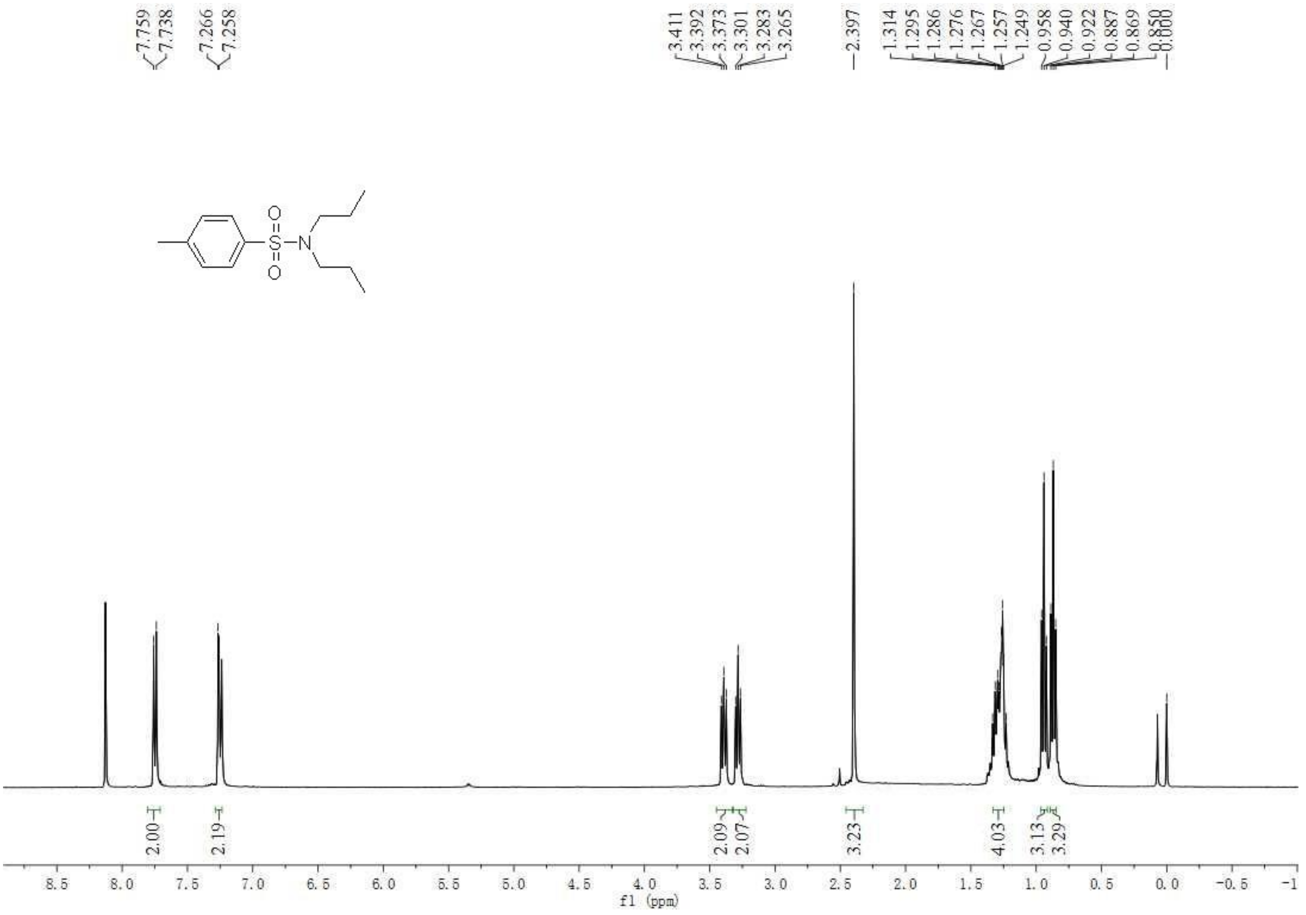

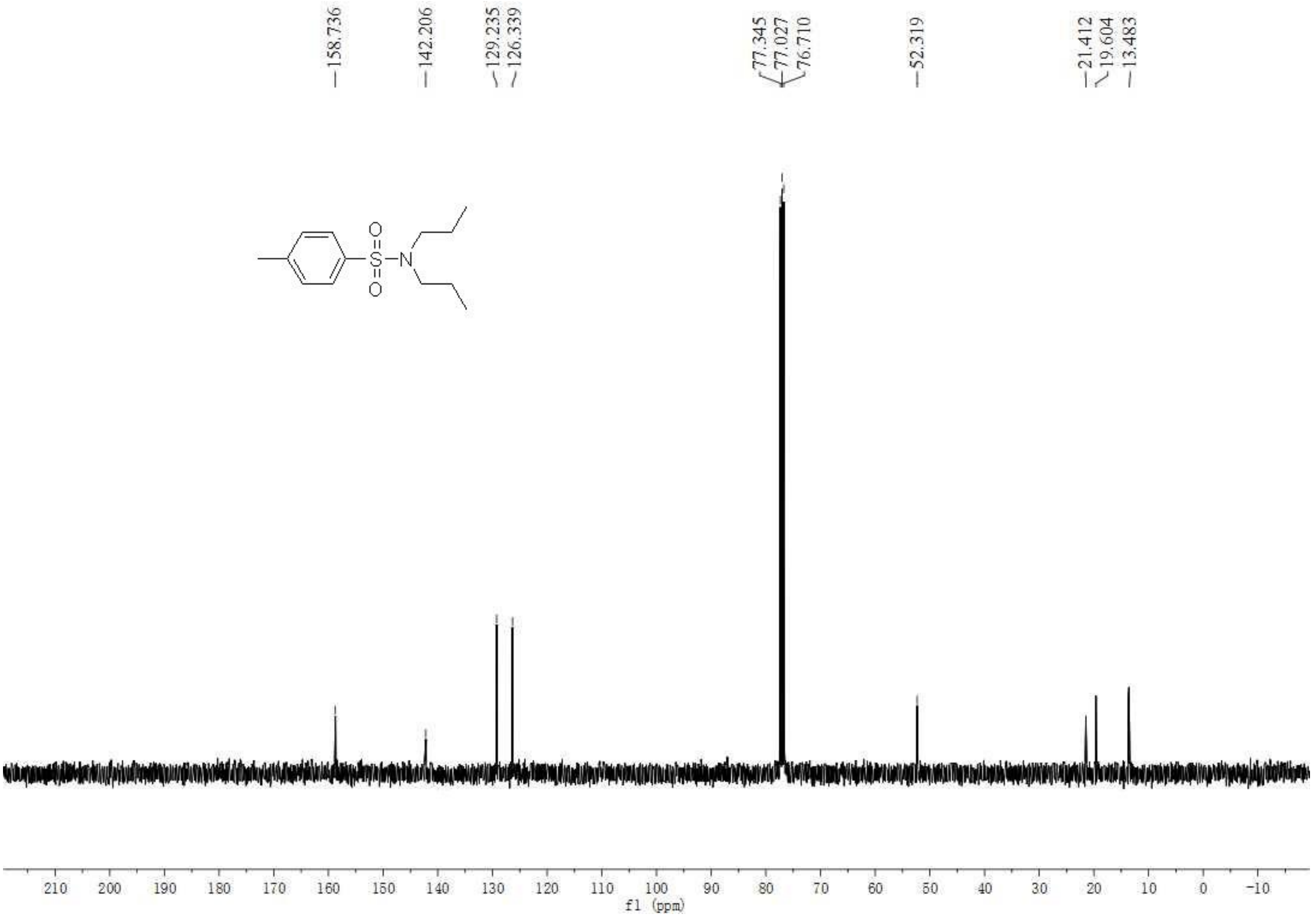

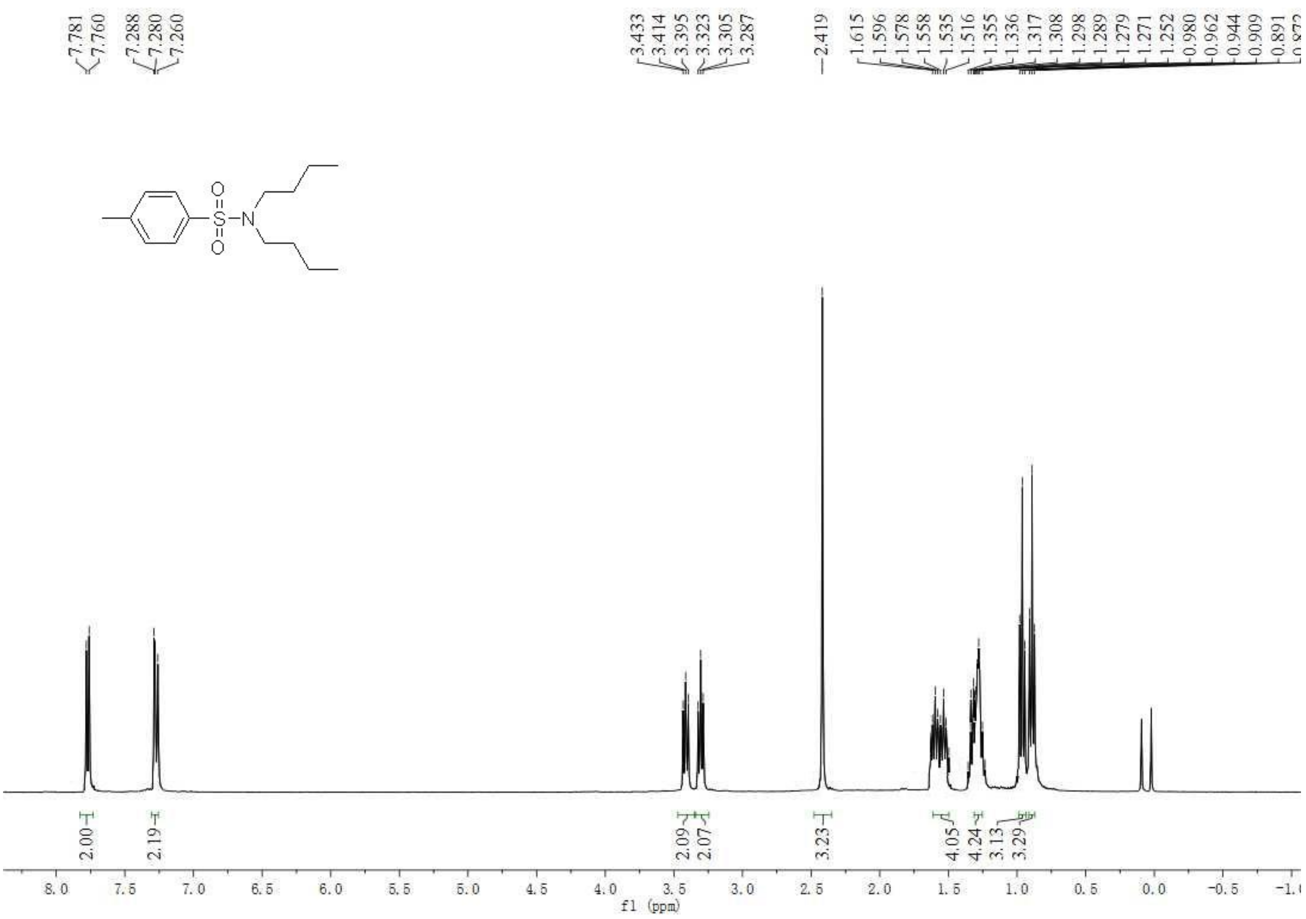
7.781
7.760
7.288
7.280
7.260
3.433
3.414
3.395
3.323
3.305
3.287
2.419
1.615
1.596
1.578
1.558
1.535
1.516
1.355
1.336
1.317
1.308
1.298
1.289
1.279
1.271
1.252
0.980
0.962
0.944
0.909
0.891
2.00
2.19
2.09
2.07
3.23
4.05
4.24
3.13
3.29
8.0 7.5 7.0 6.5 6.0 5.5 5.0 4.5 4.0 3.5 3.0 2.5 2.0 1.5 1.0 0.5 0.0 -0.5
f1 (ppm)

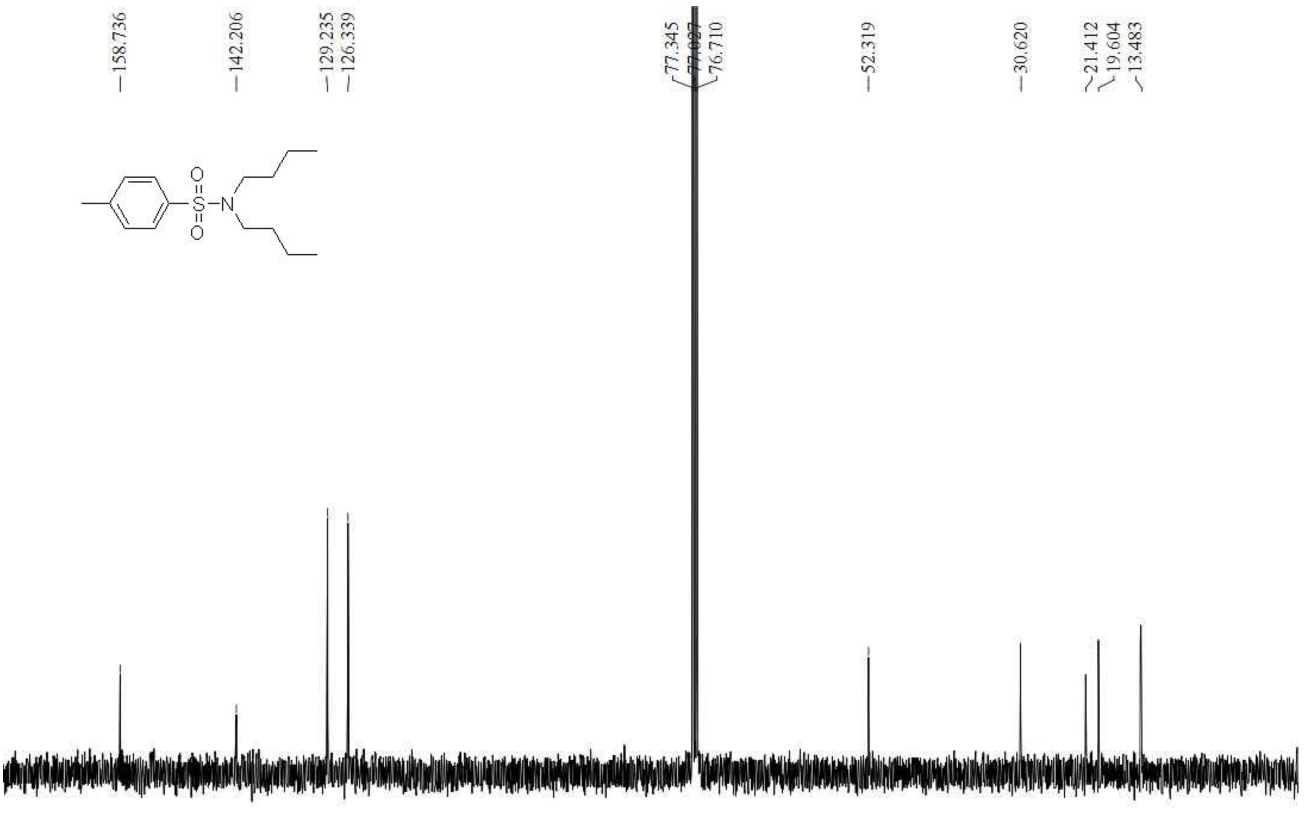
158.736
142.206
129.235
126.339
77.345
76.710
52.319
30.620
21.412
19.604
13.483
170 160 150 140 130 120 110 100 90 80 70 60 50 40 30 20 10 0
f1 (ppm)

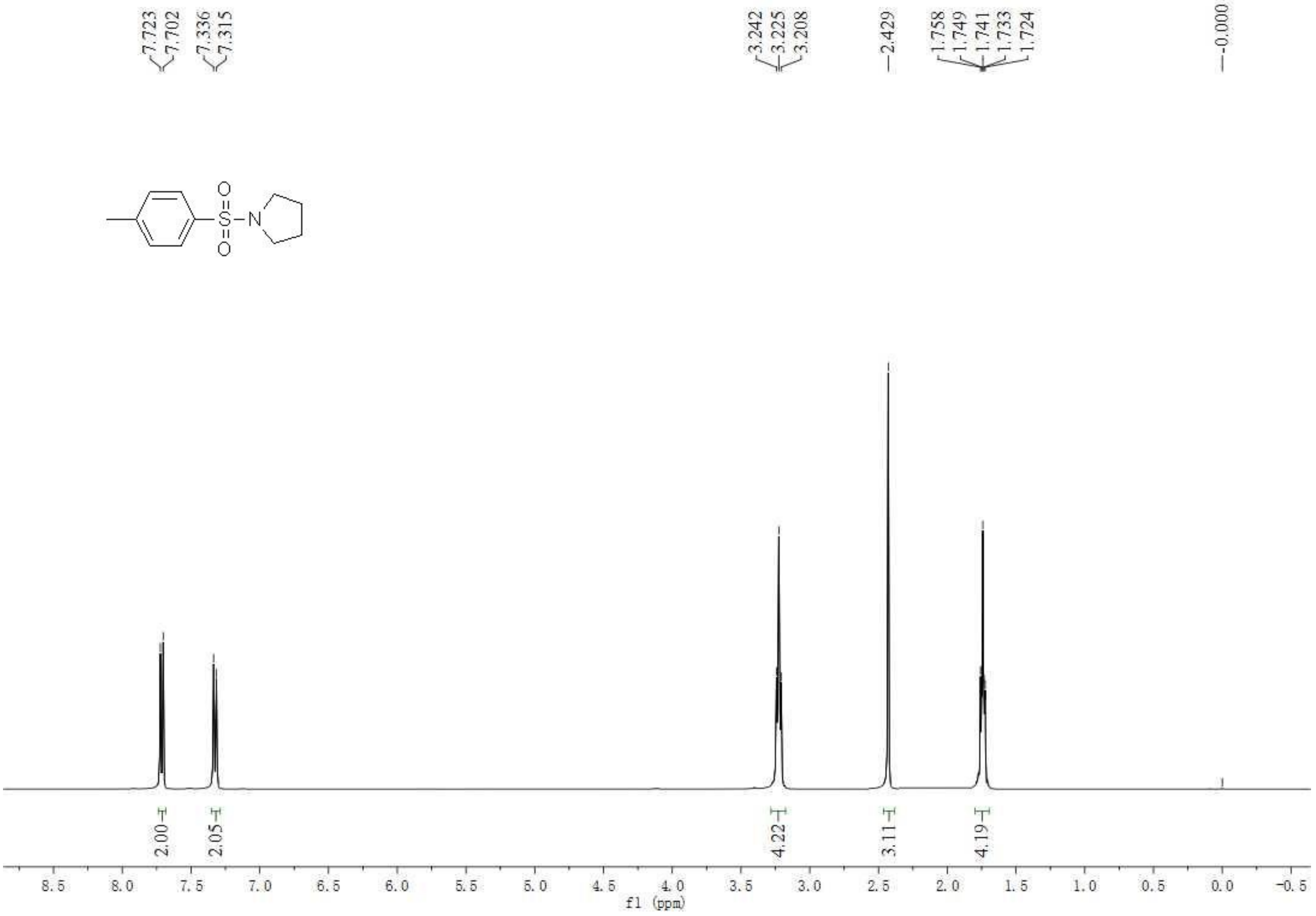
7.723
7.702
7.336
7.315
3.242
3.225
3.208
2.429
1.758
1.749
1.741
1.733
1.724
-0.000
O
S
N
O
2.00
2.05
4.22
3.11
4.19
8.5 8.0 7.5 7.0 6.5 6.0 5.5 5.0 4.5 4.0 3.5 3.0 2.5 2.0 1.5 1.0 0.5 0.0 -0.5
f1 (ppm)

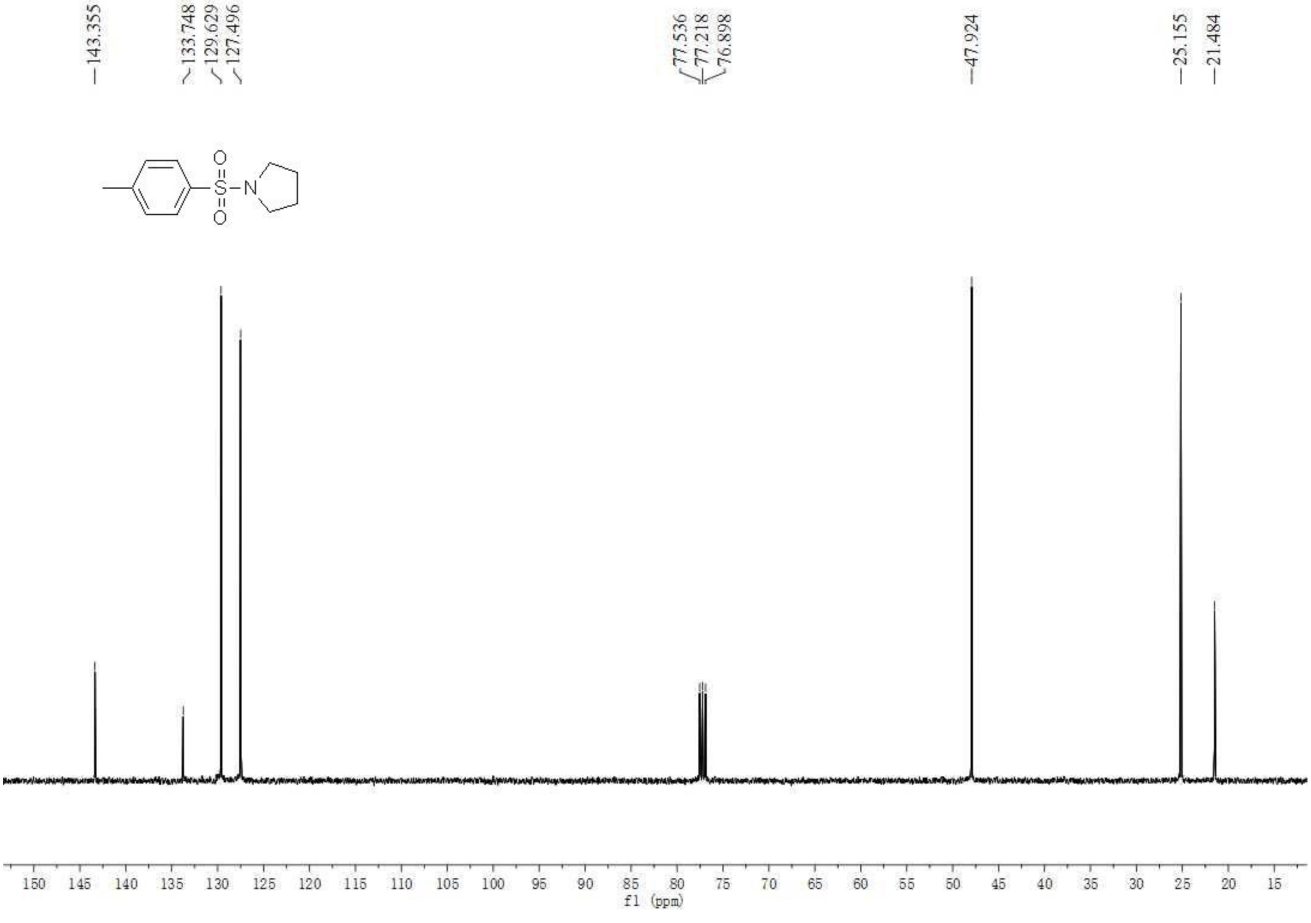
143.355
133.748
129.629
127.496
77.536
77.218
76.898
47.924
25.155
21.484
O
S
N
O
150 145 140 135 130 125 120 115 110 105 100 95 90 85 80 75 70 65 60 55 50 45 40 35 30 25 20 15
f1 (ppm)

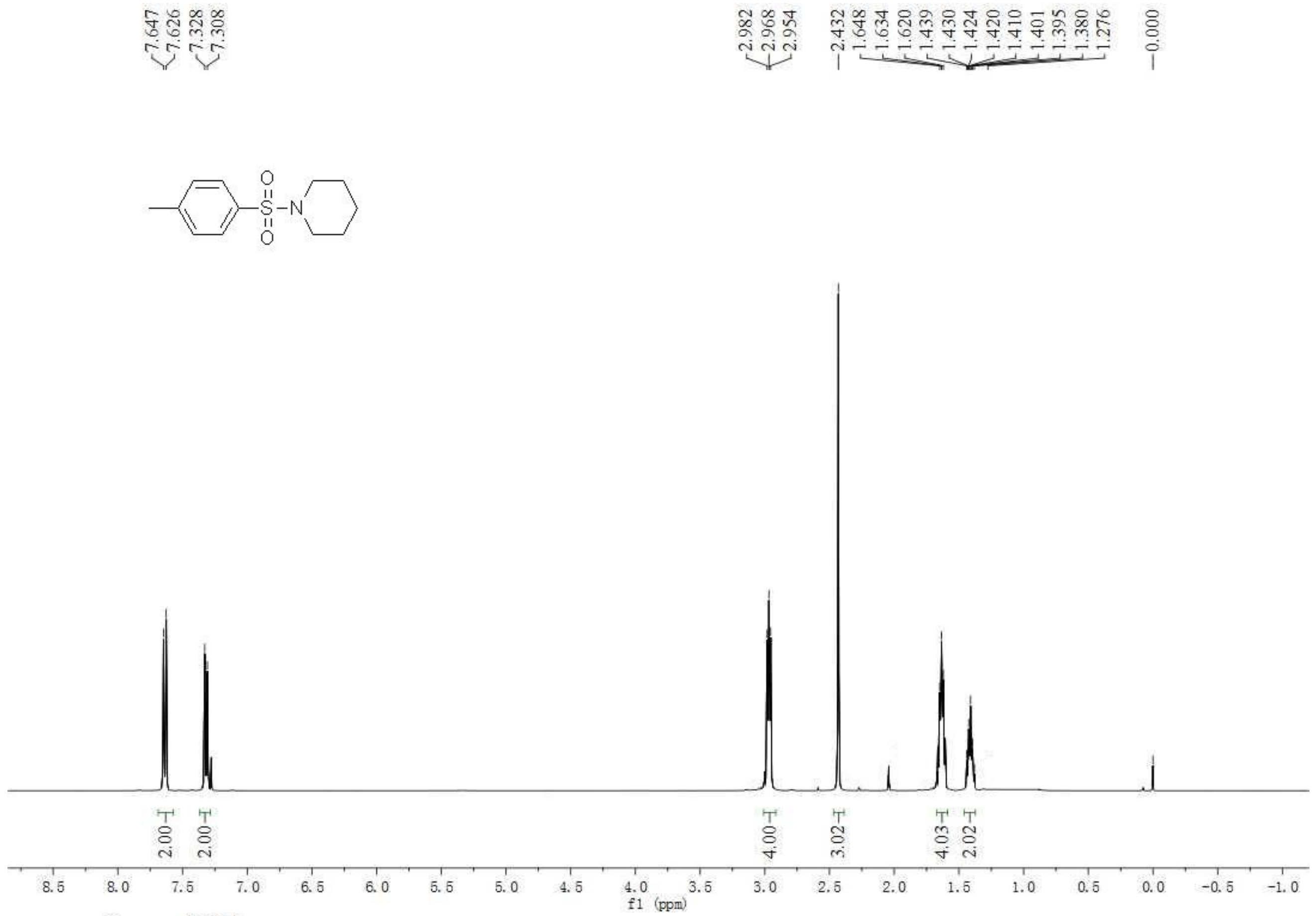

7.647
7.626
7.328
7.308
2.982
2.968
2.954
2.432
1.648
1.634
1.620
1.439
1.430
1.424
1.420
1.410
1.401
1.395
1.380
1.276
0.000
2.00
2.00
4.00
3.02
4.03
2.02
f1 (ppm)

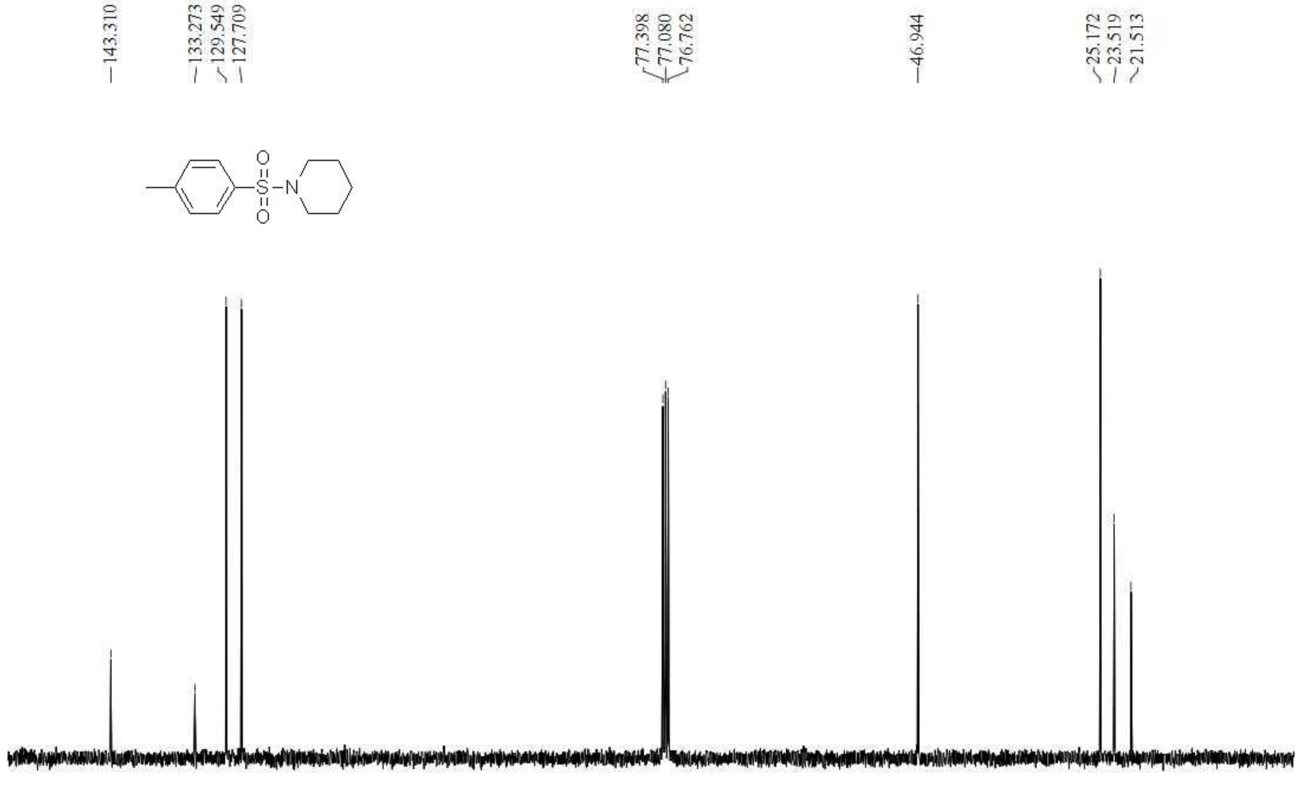

143.310
133.273
129.549
127.709
77.398
77.080
76.762
46.944
25.172
23.519
21.513
f1 (ppm)

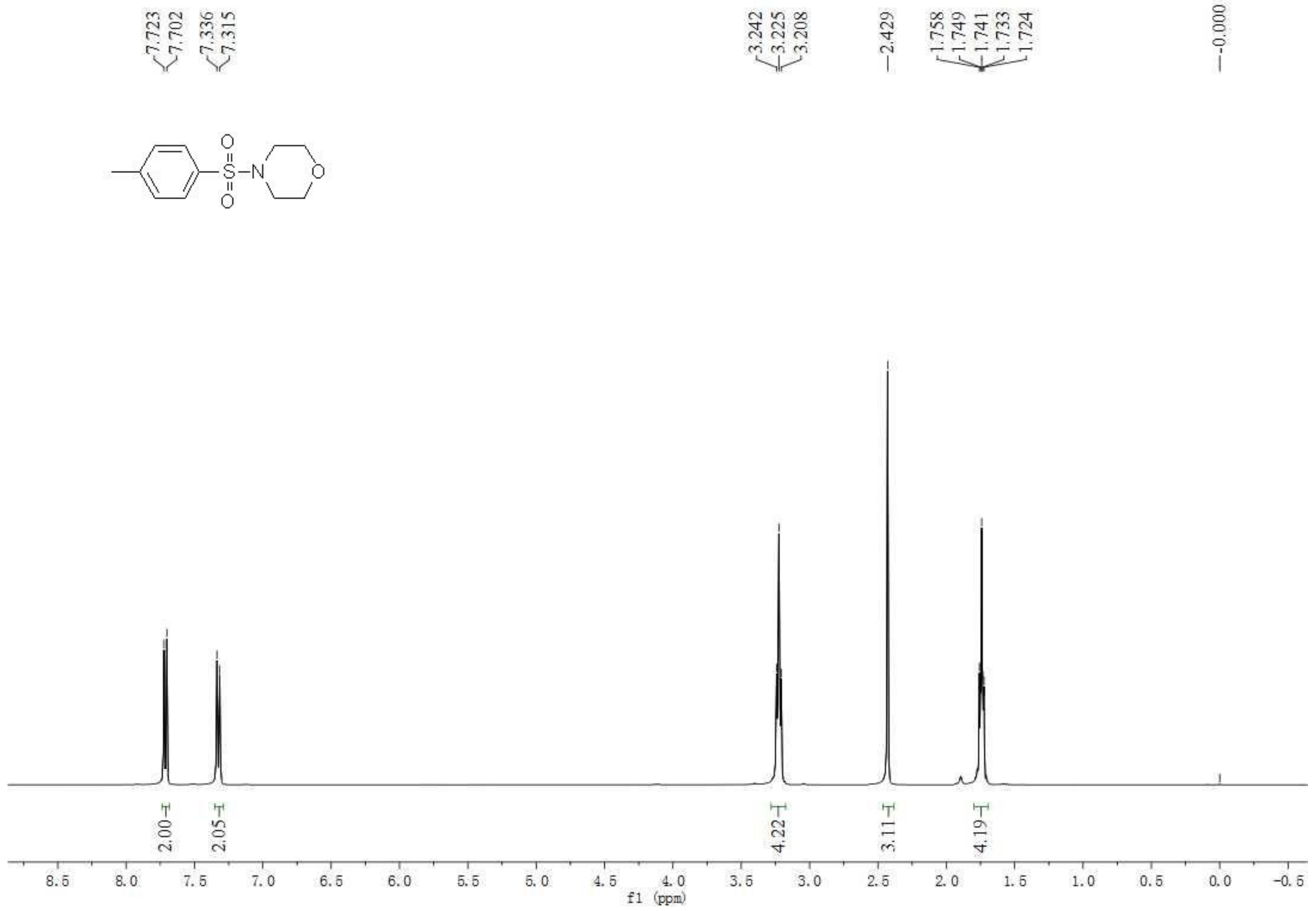
7.723
7.702
7.336
7.315
3.242
3.225
3.208
2.429
1.758
1.749
1.741
1.733
1.724
0.000
2.00
2.05
4.22
3.11
4.19
f1 (ppm)

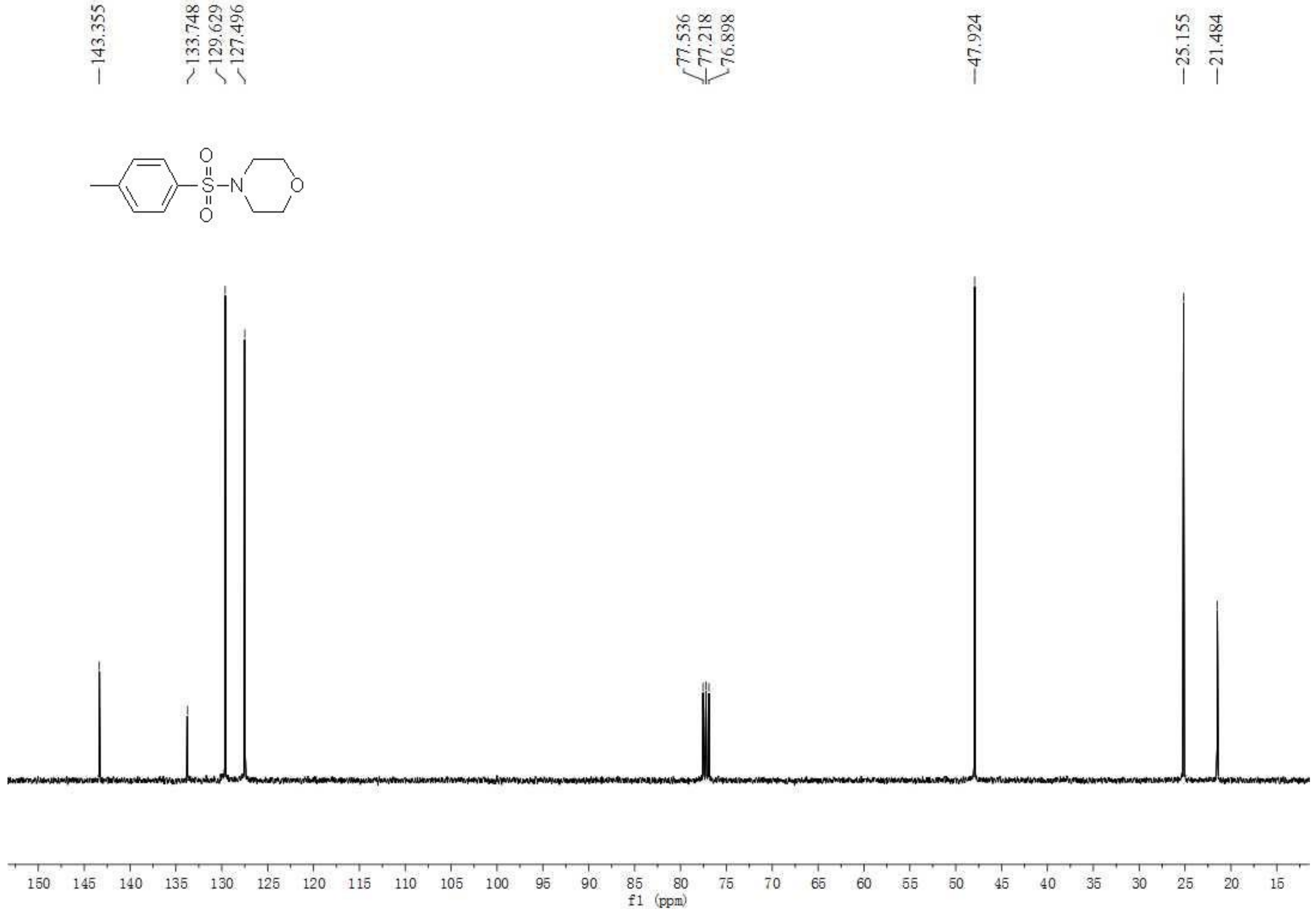
143.355
133.748
129.629
127.496
77.536
77.218
76.898
47.924
25.155
21.484
f1 (ppm)

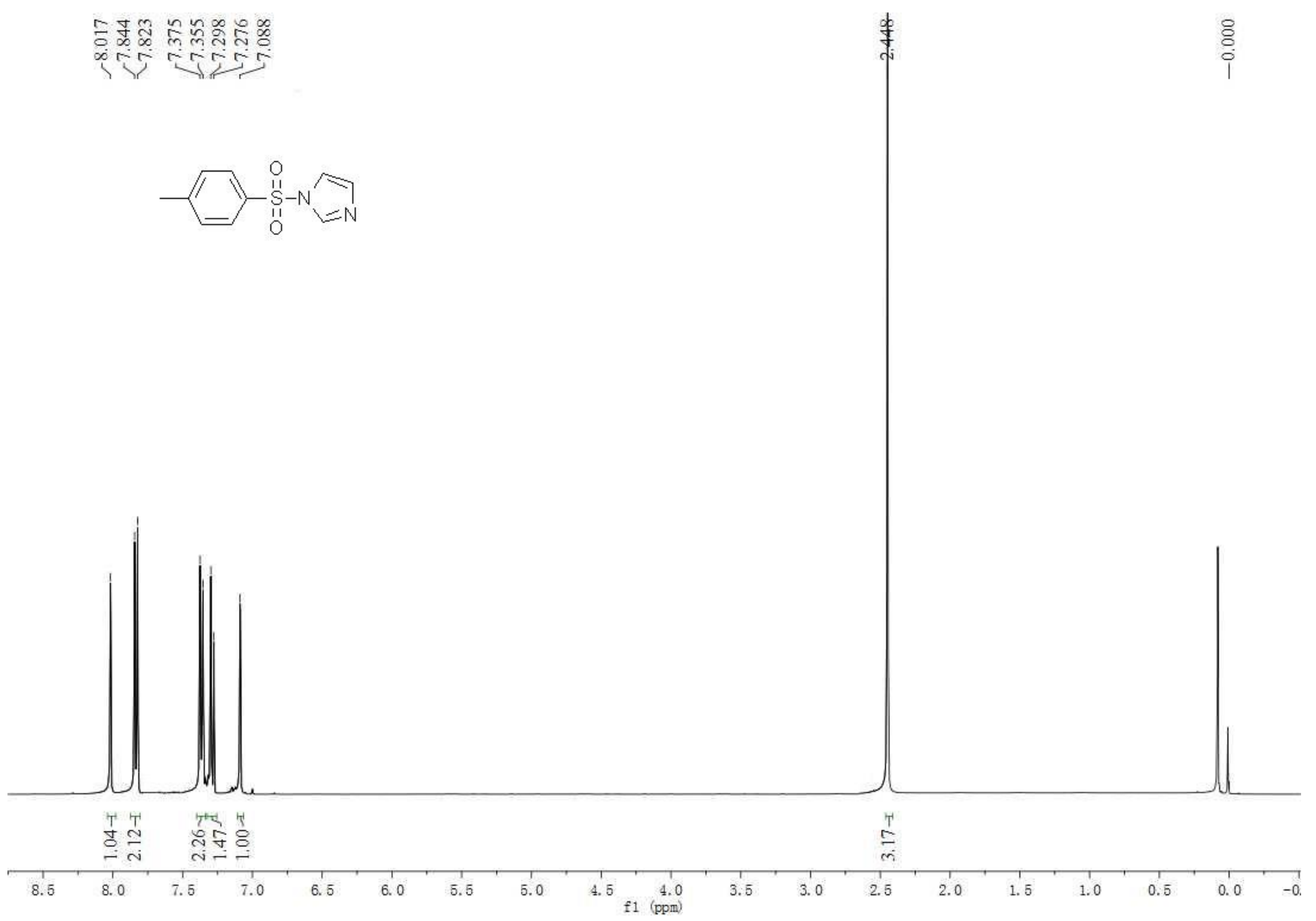
8.017
7.844
7.823
7.375
7.355
7.298
7.276
7.088
2.448
0.000
1.04
2.12
2.26
1.47
1.00
3.17
f1 (ppm)

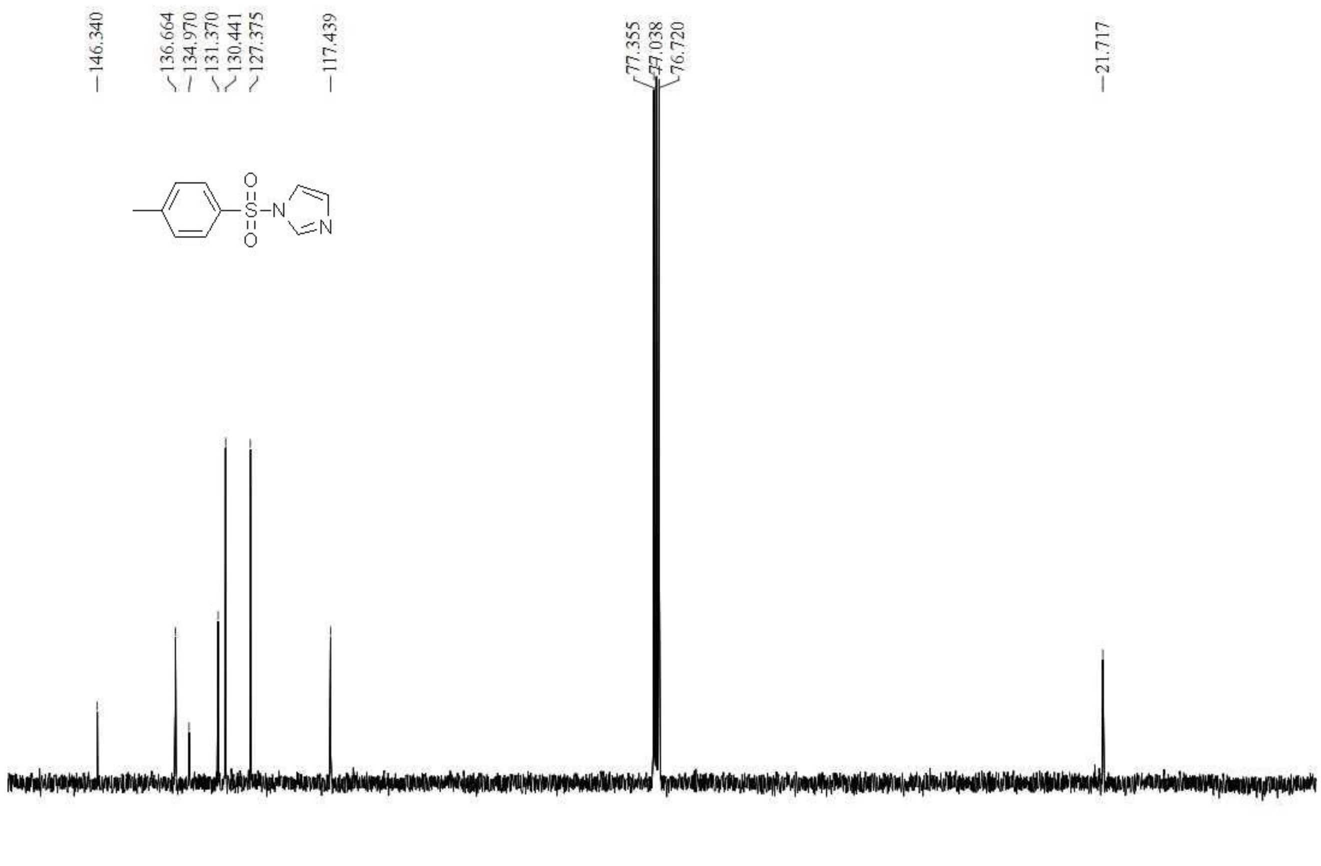
146.340
136.664
134.970
131.370
130.441
127.375
117.439
77.355
77.038
76.720
21.717
f1 (ppm)

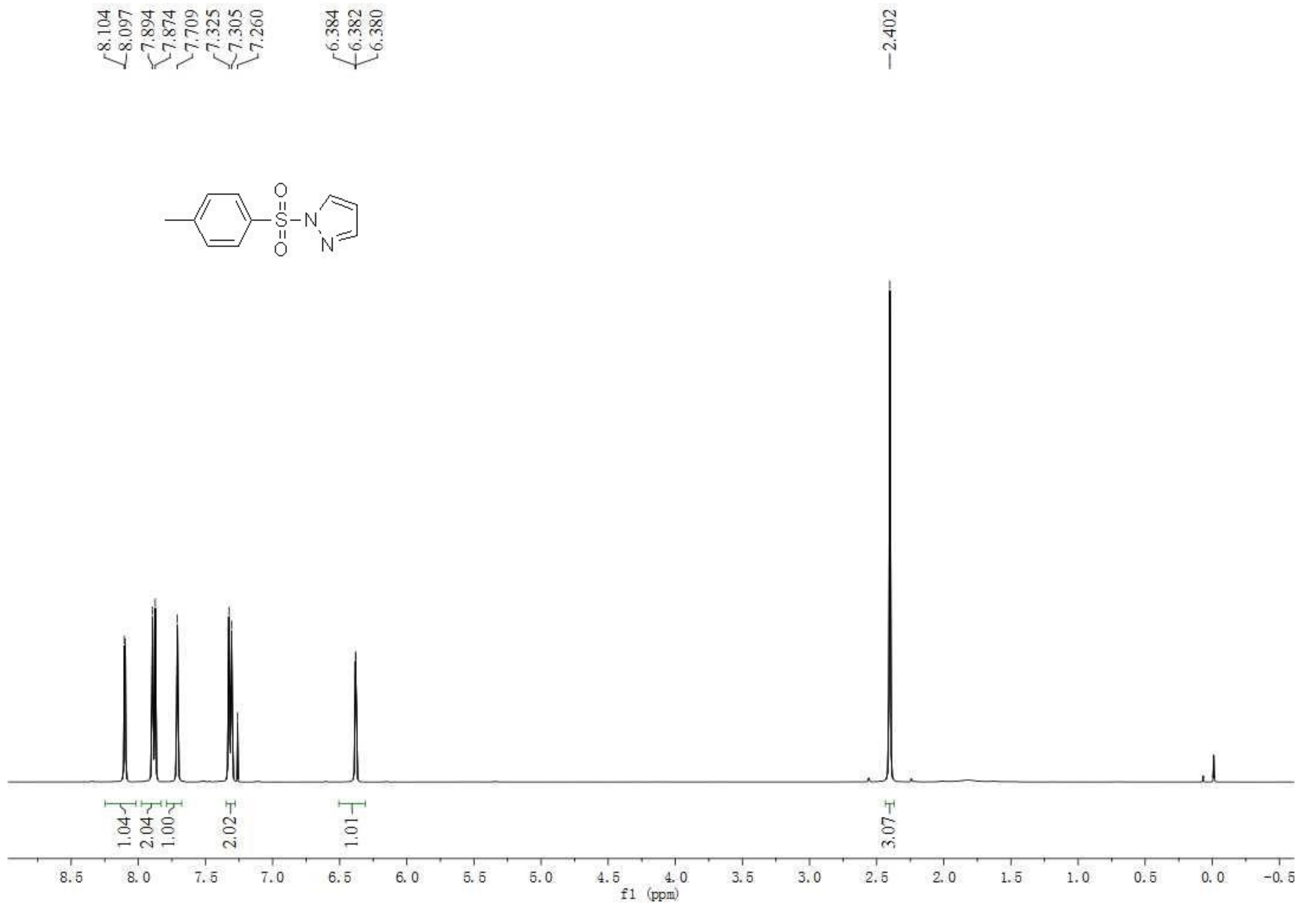
8.104
8.097
7.894
7.874
7.709
7.325
7.305
7.260
6.384
6.382
6.380
2.402
1.04
2.04
1.00
2.02
1.01
3.07
f1 (ppm)

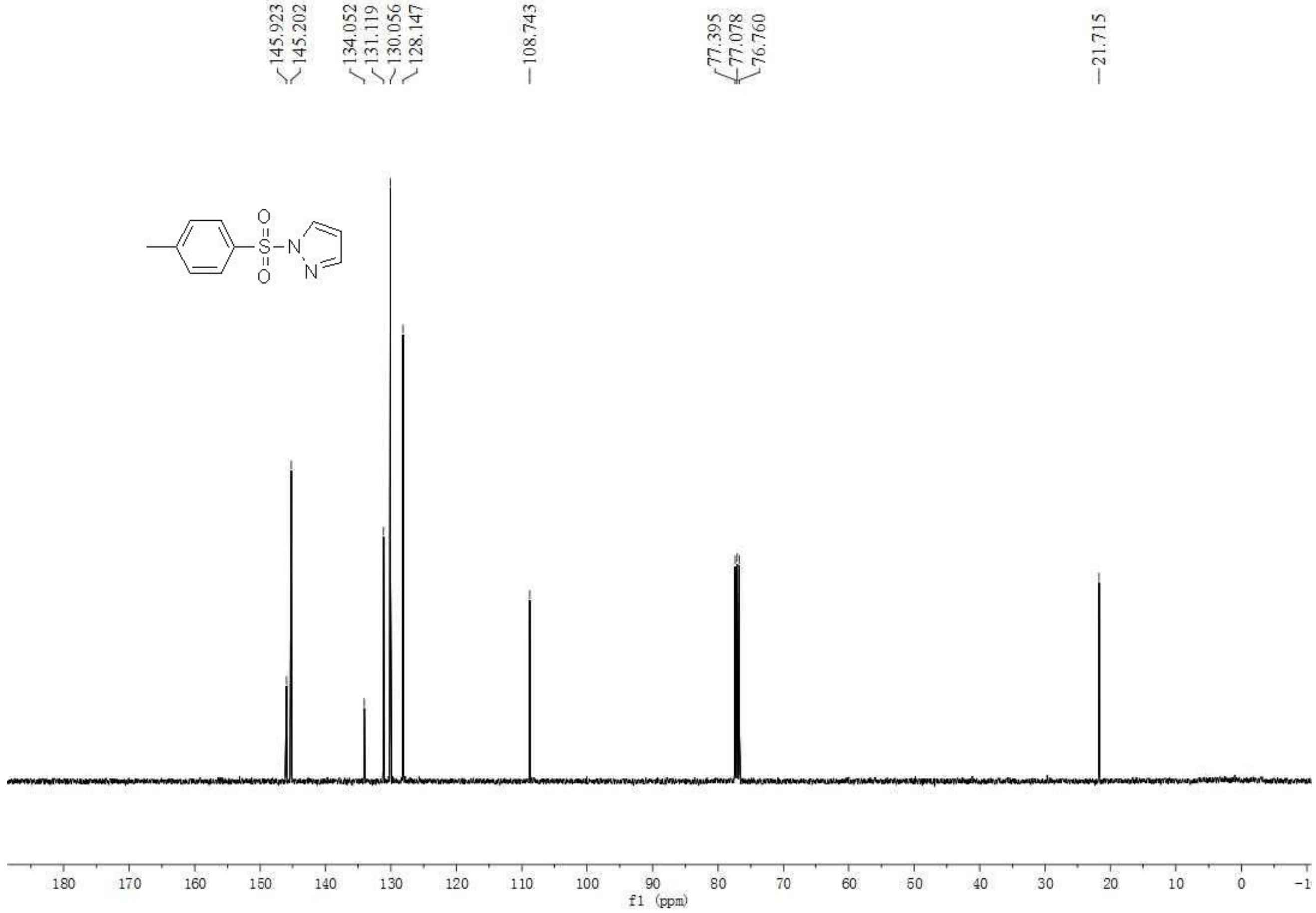
145.923
145.202
134.052
131.119
130.056
128.147
108.743
77.395
77.078
76.760
21.715
f1 (ppm)

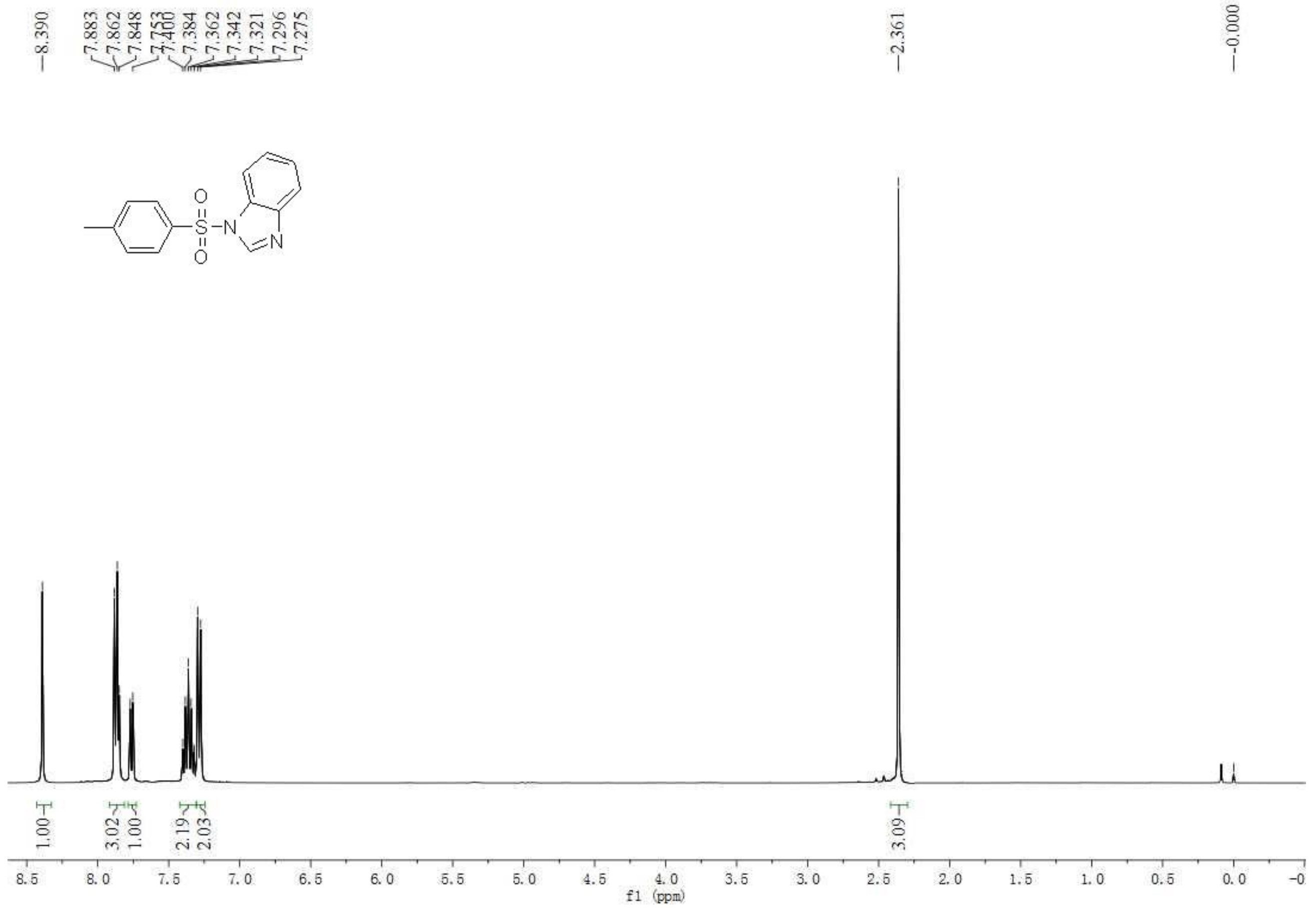
8.390
7.883
7.862
7.848
7.753
7.400
7.384
7.362
7.342
7.321
7.296
7.275
2.361
0.000
1.00
3.02
1.00
2.19
2.03
3.09
f1 (ppm)

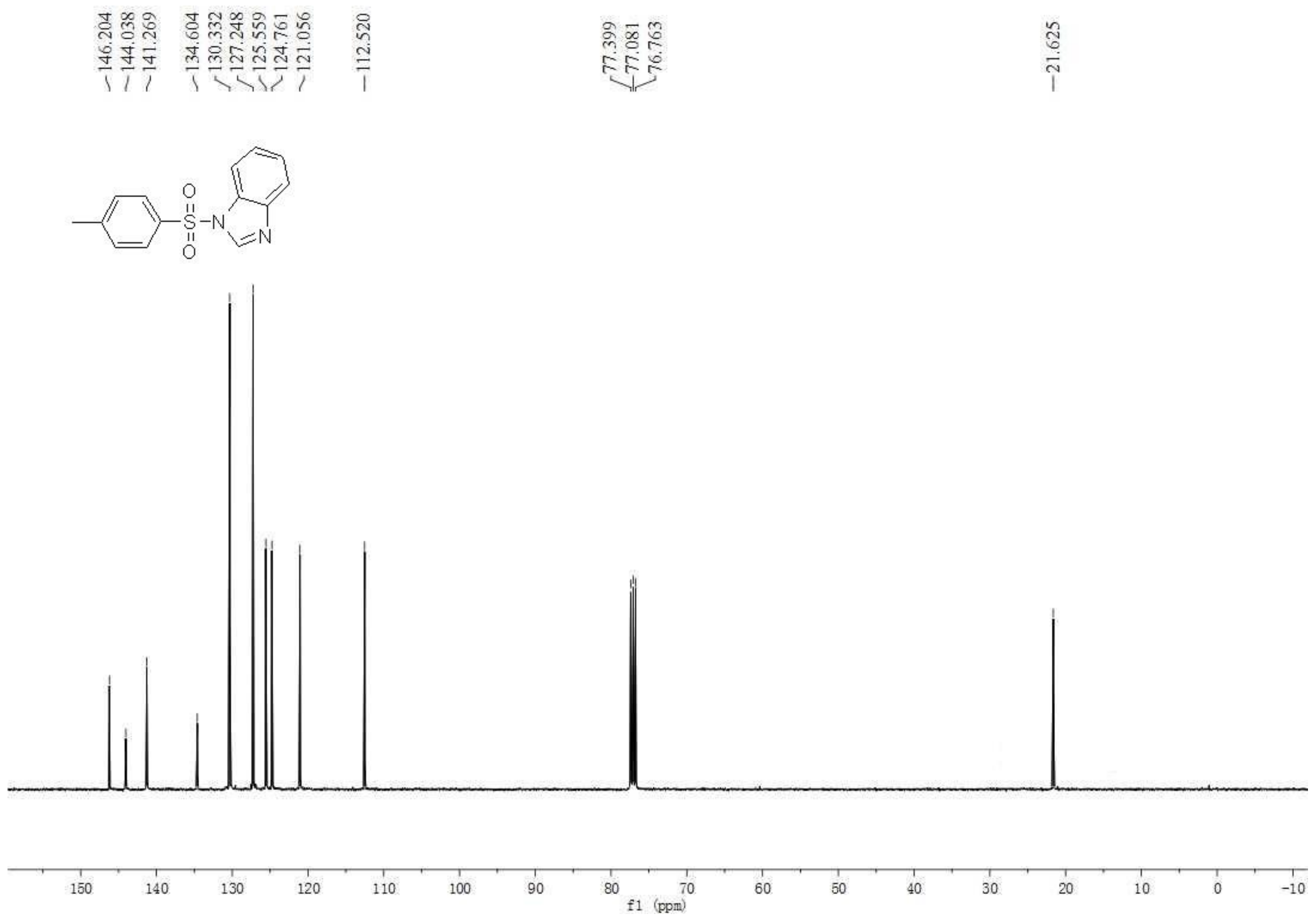
146.204
144.038
141.269
134.604
130.332
127.248
125.559
124.761
121.056
112.520
77.399
77.081
76.763
21.625
f1 (ppm)

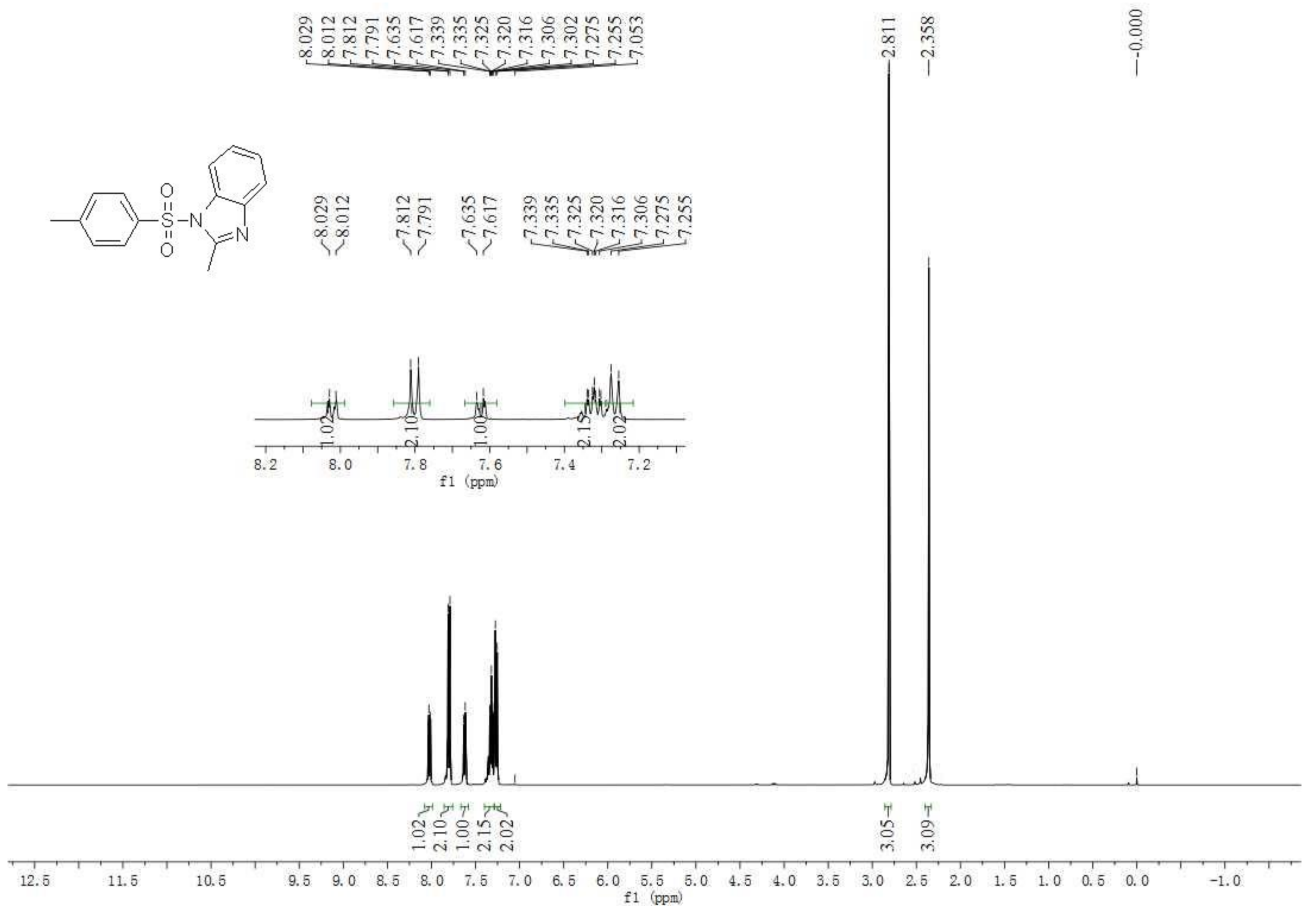
8.029
8.012
7.812
7.791
7.635
7.617
7.339
7.335
7.325
7.320
7.316
7.306
7.302
7.275
7.255
7.053
2.811
2.358
0.000
1.02
2.10
1.00
2.15
2.02
3.05
3.09
f1 (ppm)

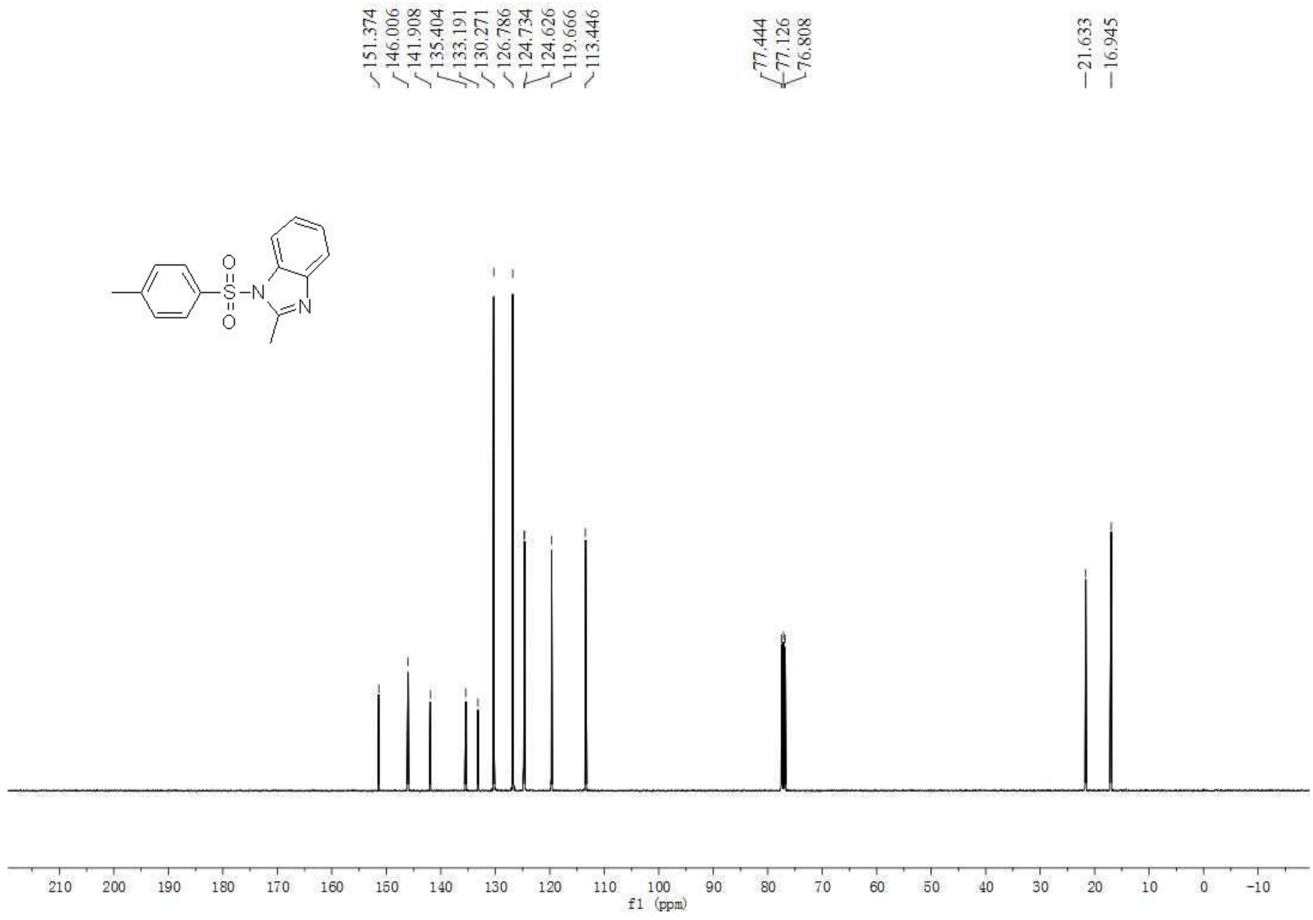
151.374
146.006
141.908
135.404
133.191
130.271
126.786
124.734
124.626
119.666
113.446
77.444
77.126
76.808
21.633
16.945
f1 (ppm)

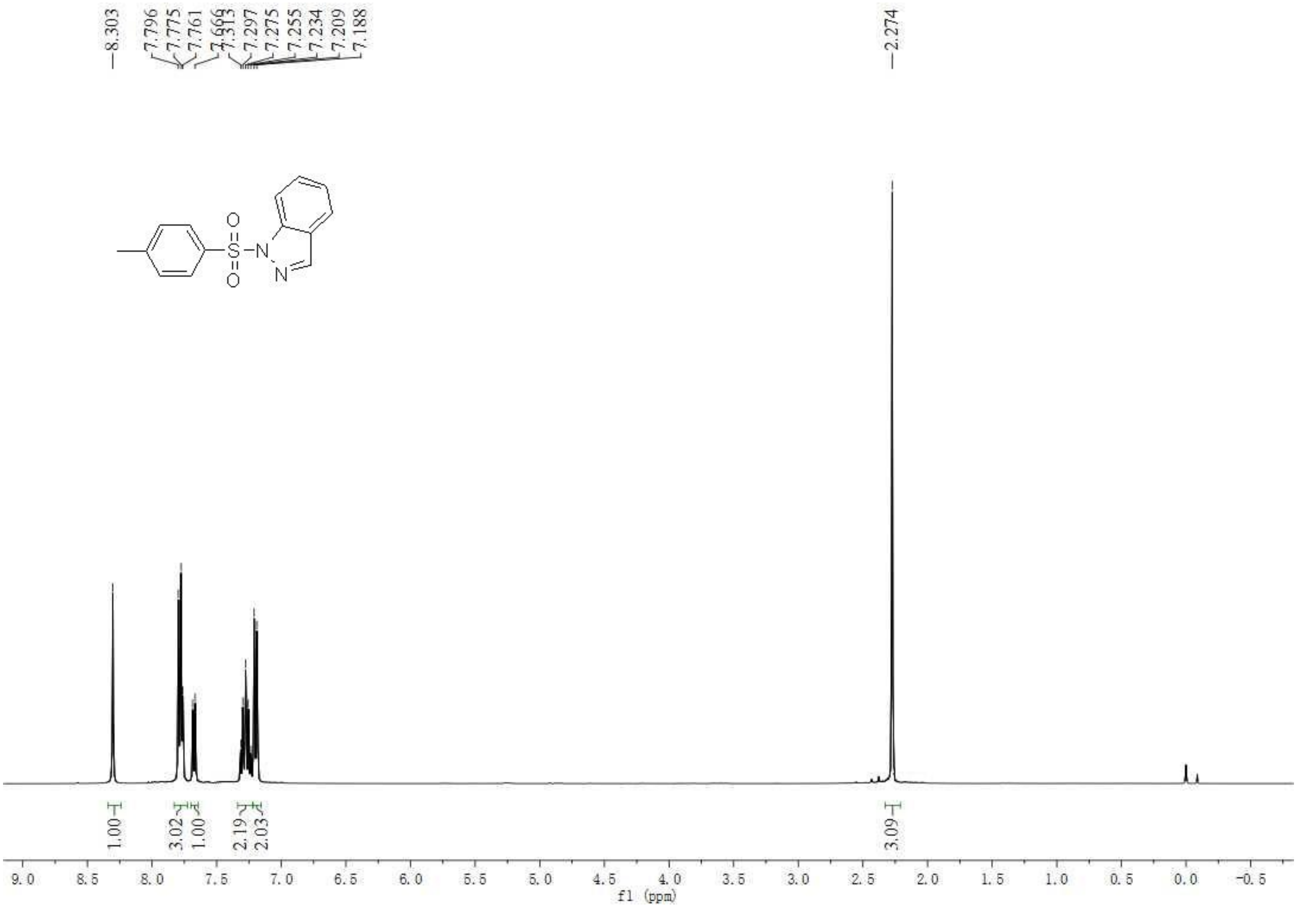
8.303
7.796
7.775
7.761
7.666
7.313
7.297
7.275
7.255
7.234
7.209
7.188
2.274
1.00
3.02
1.00
2.19
2.03
3.09
f1 (ppm)

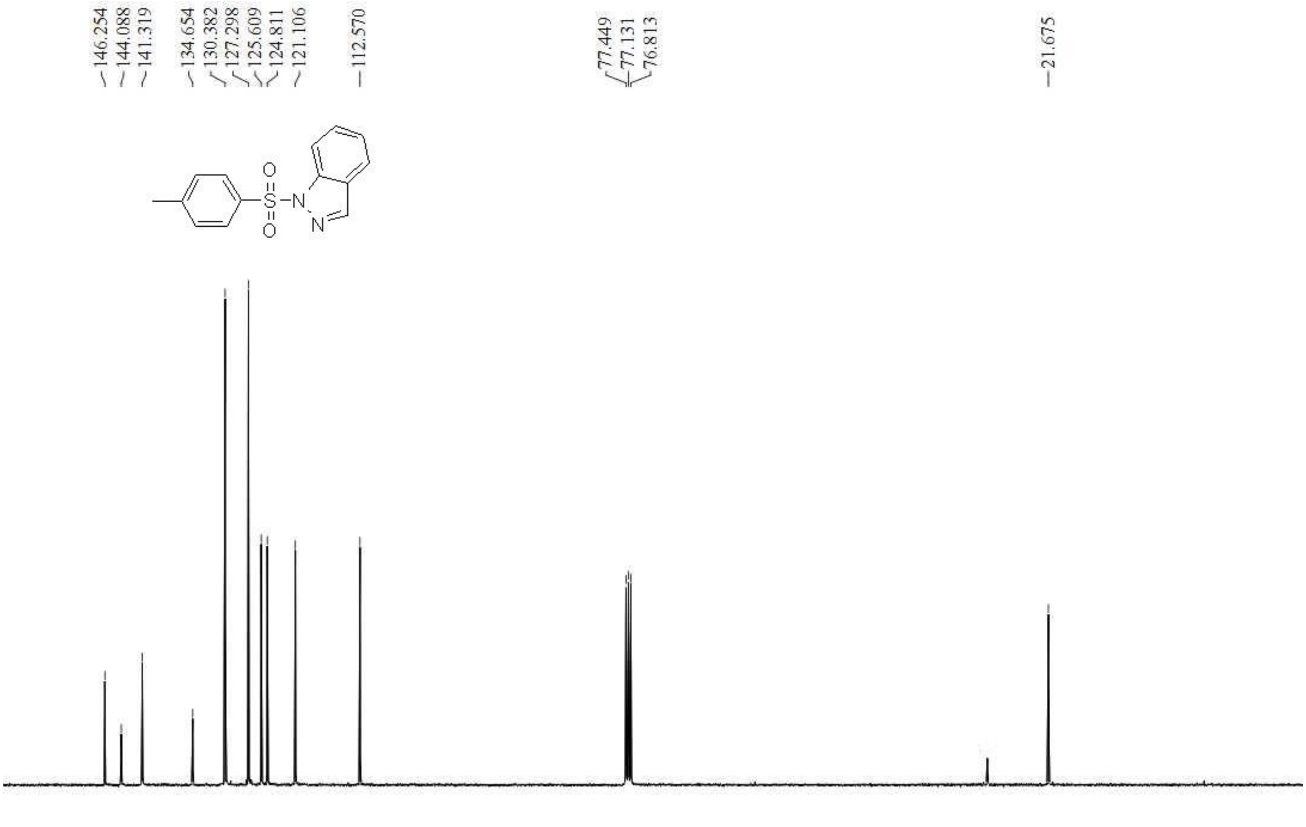
146.254
144.088
141.319
134.654
130.382
127.298
125.609
124.811
121.106
112.570
77.449
77.131
76.813
21.675
f1 (ppm)

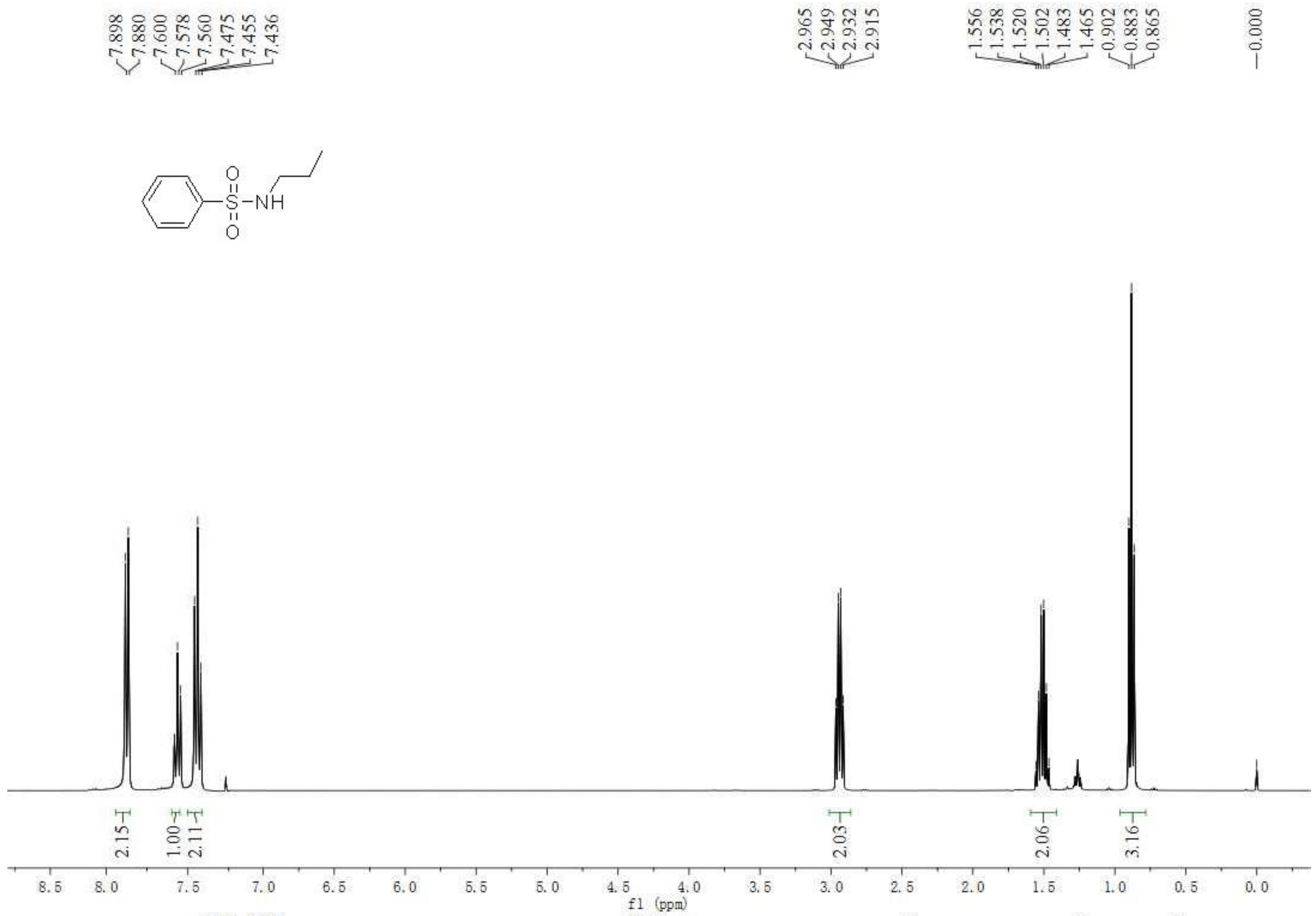
7.898
7.880
7.600
7.578
7.560
7.475
7.455
7.436
2.965
2.949
2.932
2.915
1.556
1.538
1.520
1.502
1.483
1.465
0.902
0.883
0.865
0.000
NH
2.15
1.00
2.11
2.03
2.06
3.16
8.5 8.0 7.5 7.0 6.5 6.0 5.5 5.0 4.5 4.0 3.5 3.0 2.5 2.0 1.5 1.0 0.5 0.0
f1 (ppm)

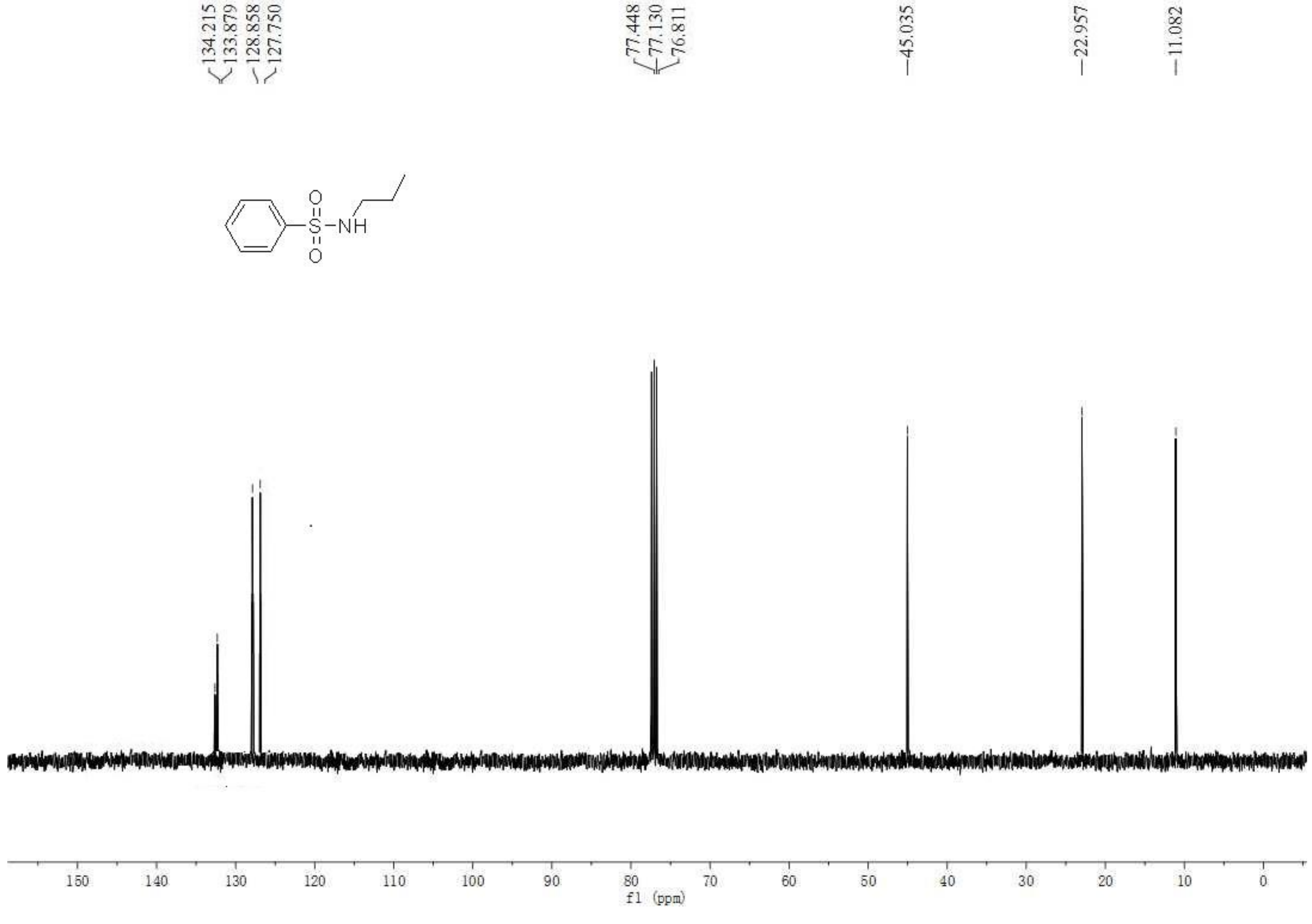
134.215
133.879
128.858
127.750
77.448
77.130
76.811
45.035
22.957
11.082
NH
150 140 130 120 110 100 90 80 70 60 50 40 30 20 10 0
f1 (ppm)

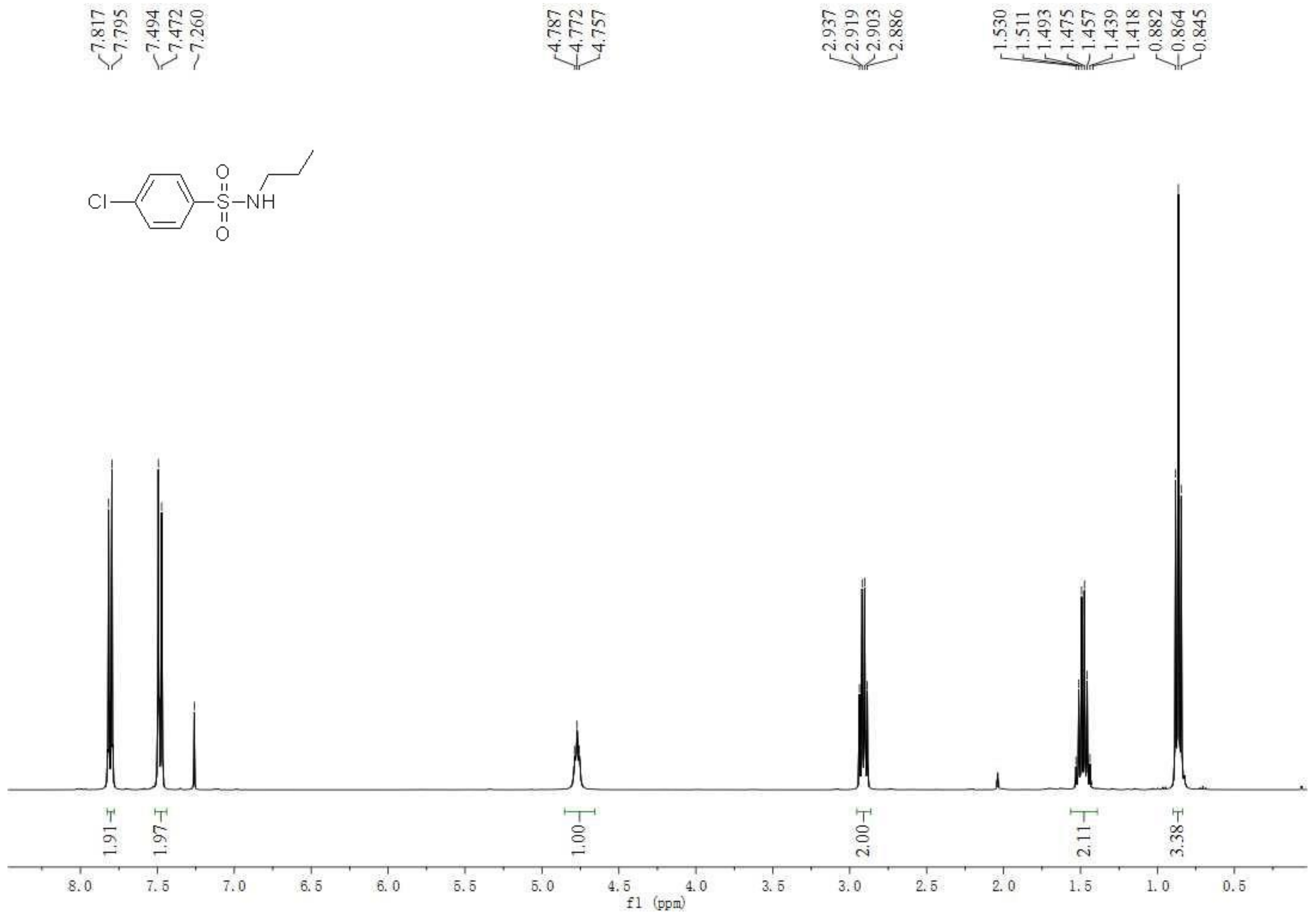
7.817
7.795
7.494
7.472
7.260
4.787
4.772
4.757
2.937
2.919
2.903
2.886
1.530
1.511
1.493
1.475
1.457
1.439
1.418
0.882
0.864
0.845
Cl
S
O
O
NH
1.91
1.97
1.00
2.00
2.11
3.38
8.0
7.5
7.0
6.5
6.0
5.5
5.0
4.5
4.0
3.5
3.0
2.5
2.0
1.5
1.0
0.5
f1 (ppm)
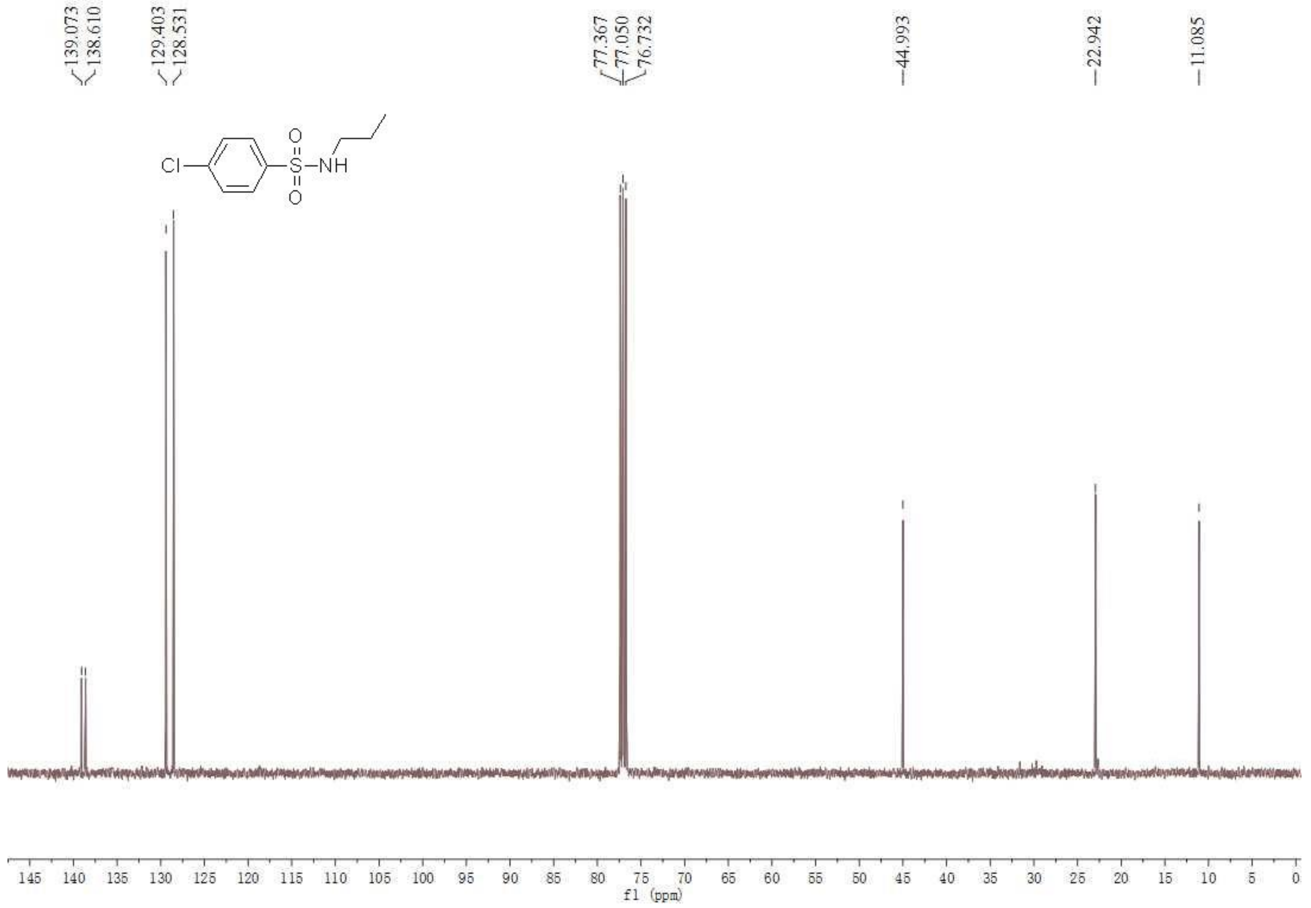
139.073
138.610
129.403
128.531
77.367
77.050
76.732
44.993
22.942
11.085
Cl
S
O
O
NH
145
140
135
130
125
120
115
110
105
100
95
90
85
80
75
70
65
60
55
50
45
40
35
30
25
20
15
10
5
0
f1 (ppm)

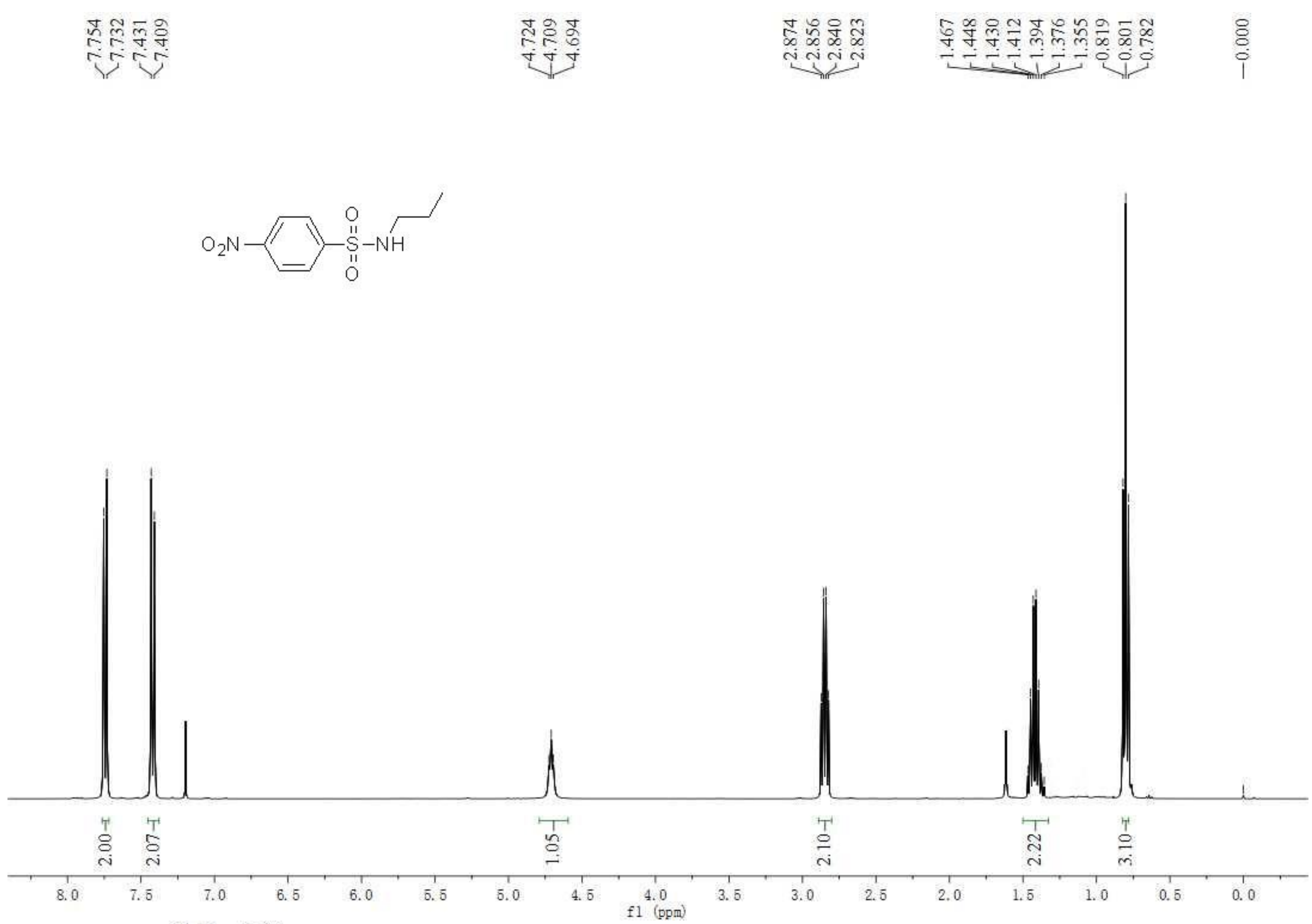
7.754
7.732
7.431
7.409
4.724
4.709
4.694
2.874
2.856
2.840
2.823
1.467
1.448
1.430
1.412
1.394
1.376
1.355
0.819
0.801
0.782
0.000
O2N
S
O
O
NH
2.00
2.07
1.05
2.10
2.22
3.10
8.0
7.5
7.0
6.5
6.0
5.5
5.0
4.5
4.0
3.5
3.0
2.5
2.0
1.5
1.0
0.5
0.0
f1 (ppm)

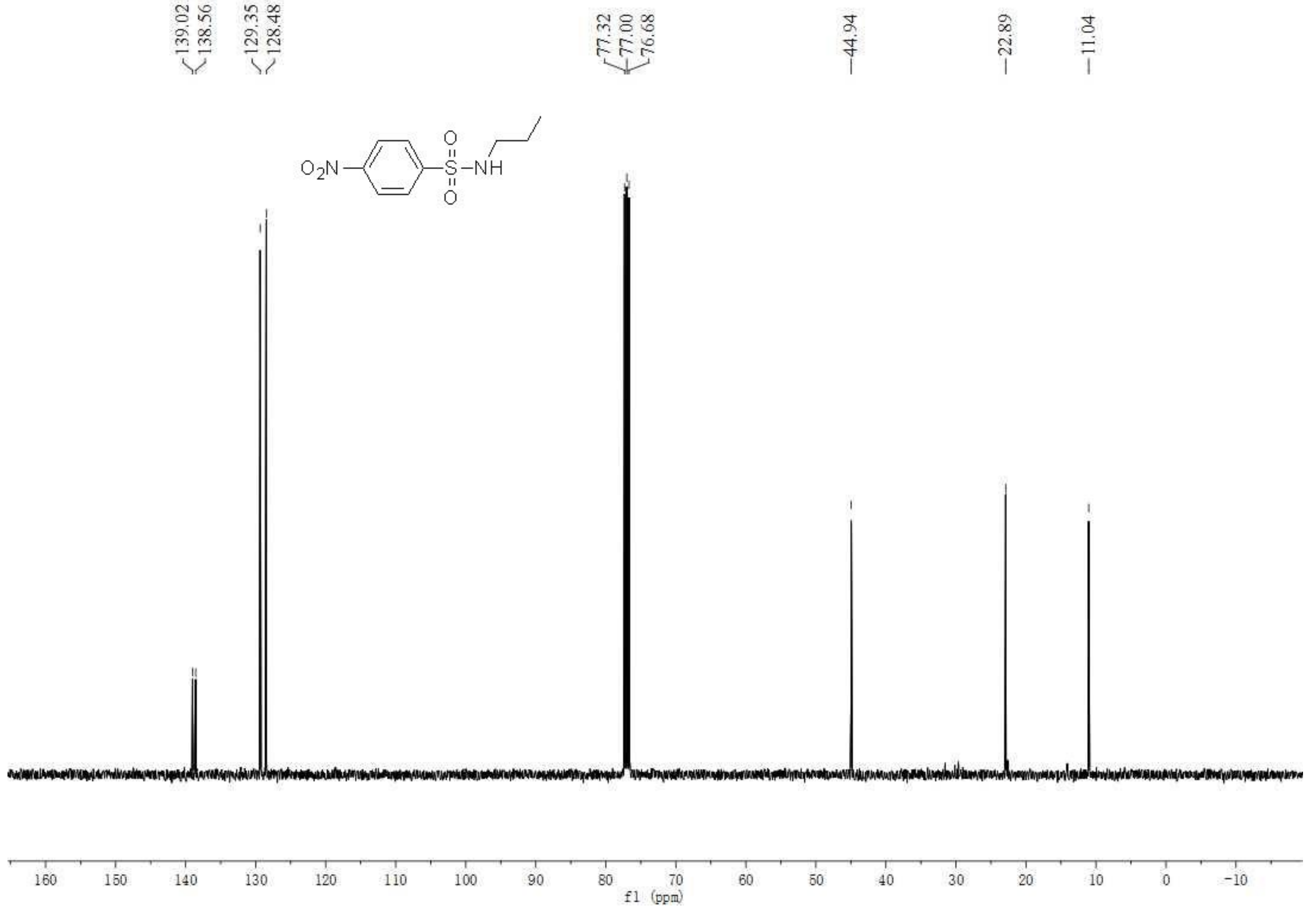
139.02
138.56
129.35
128.48
77.32
77.00
76.68
44.94
22.89
11.04
O2N
S
O
O
NH
160
150
140
130
120
110
100
90
80
70
60
50
40
30
20
10
0
-10
f1 (ppm)

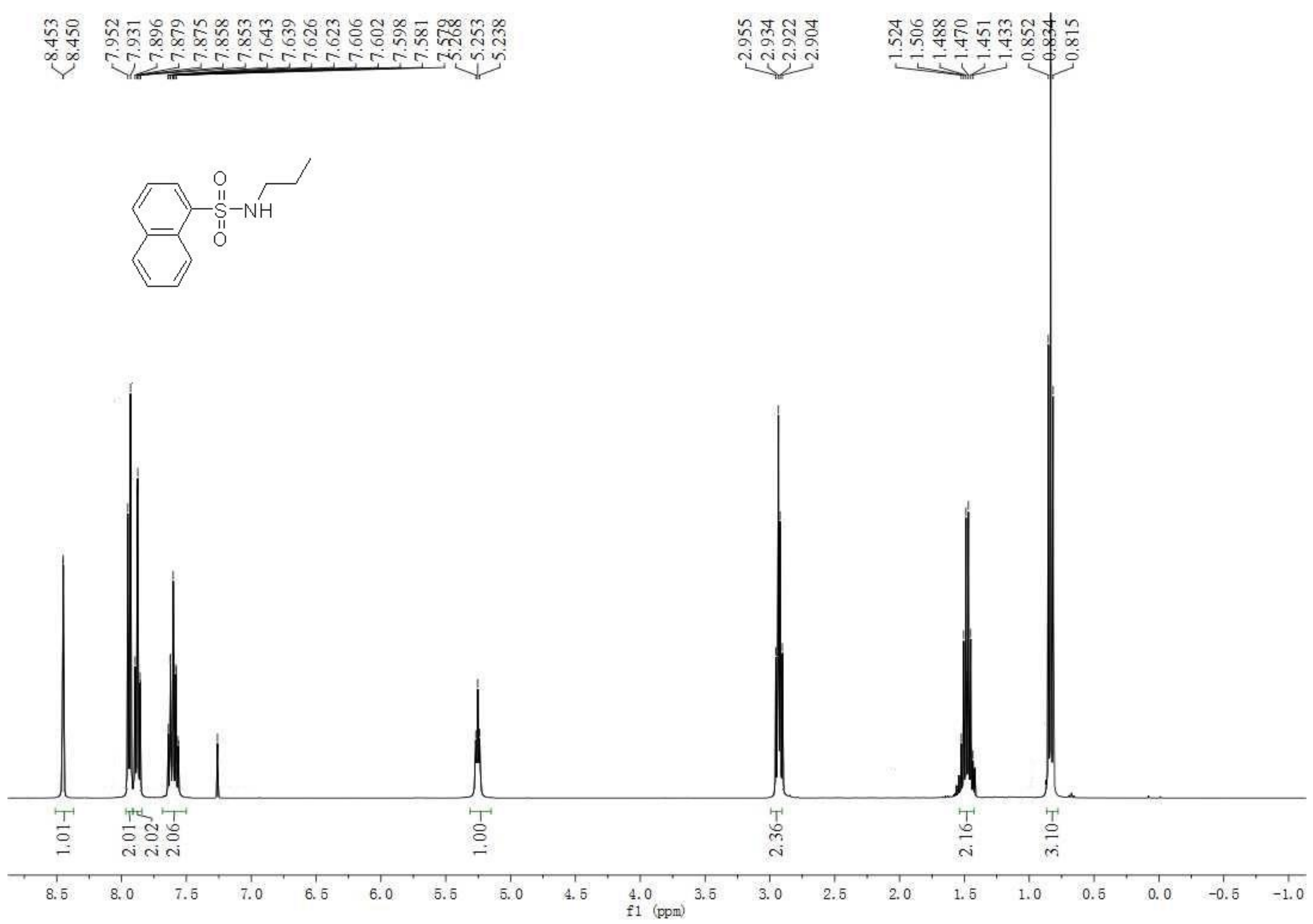

8.453
8.450
7.952
7.931
7.896
7.879
7.875
7.858
7.853
7.643
7.639
7.626
7.623
7.606
7.602
7.598
7.581
7.579
5.268
5.253
5.238
2.955
2.934
2.922
2.904
1.524
1.506
1.488
1.470
1.451
1.433
0.852
0.834
0.815
NH
O
S
O
1.01
2.01
2.02
2.06
1.00
2.36
2.16
3.10
f1 (ppm)

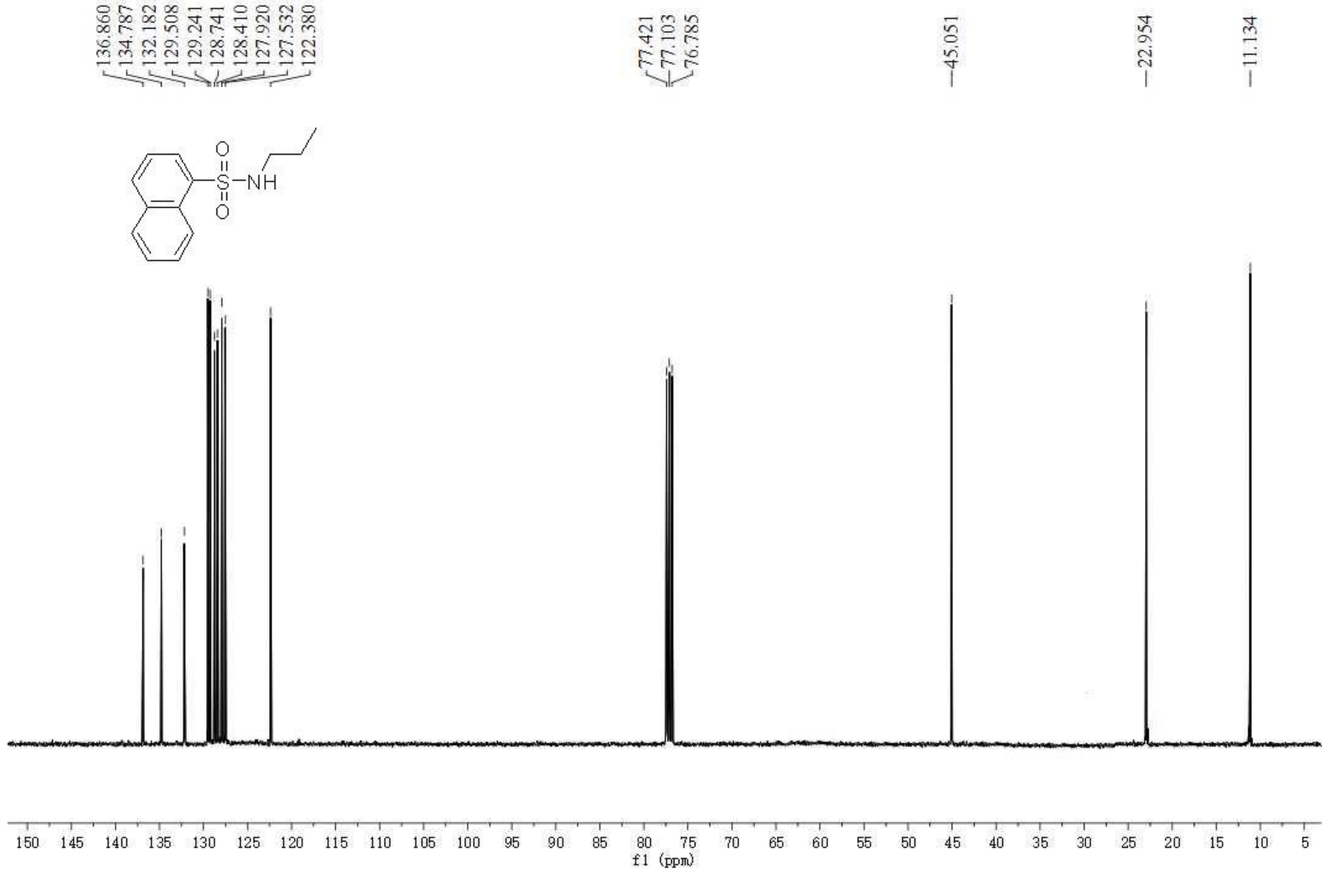

136.860
134.787
132.182
129.508
129.241
128.741
128.410
127.920
127.532
122.380
77.421
77.103
76.785
45.051
22.954
11.134
NH
O
S
O
f1 (ppm)
